# Encoding Cumulation to Learn Perturbative Nonlinear Oscillatory Dynamics

**DOI:** 10.1002/advs.202519707

**Published:** 2026-03-06

**Authors:** Teng Ma, Ting‐Ting Gao, Wei Cui, Attilio Frangi, Gang Yan, Lin Zhao

**Affiliations:** ^1^ State Key Lab of Disaster Reduction in Civil Engineering Tongji University Shanghai P. R. China; ^2^ Department of Civil and Environmental Engineering Politecnico di Milano Milan Italy; ^3^ Network Science Institute Northeastern University Boston Massachusetts USA; ^4^ MOE Key Laboratory of Advanced Micro‐Structured Materials, and School of Physical Science and Engineering Tongji University Shanghai P. R. China; ^5^ National Key Laboratory of Autonomous Intelligent Unmanned Systems, MOE Frontiers Science Center for Intelligent Autonomous Systems, and Shanghai Key Laboratory of Intelligent Autonomous Systems Tongji University Shanghai P. R. China; ^6^ Key Laboratory of Disaster Prevention and Structural Safety of China Ministry of Education Guangxi University Nanning P. R. China

**Keywords:** machine learning, model discovery, oscillatory dynamics, perturbative systems

## Abstract

Oscillatory dynamics play a central role in the description of a broad spectrum of physical systems. While often well‐approximated by linear models, the essential long‐term evolution and stability of these systems are frequently determined by subtle nonlinearities. The characterization of such weakly nonlinear systems from observational data is a central challenge in system identification, a task made difficult by the immense disparity in magnitude between faint nonlinear signatures and the dominant linear response. Herein, we introduce Evolutionary Learning Oscillator with Weak Nonlinearity (EvLOWN), a data‐driven methodology for inferring the governing equations of weakly nonlinear oscillators from sparse and potentially noisy time‐series observations. We first demonstrate EvLOWN's superior accuracy and robustness on benchmark systems, then apply it to uncover the subtle on‐site and coupling potentials in fundamental models, including the Fermi‐Pasta‐Ulam and Klein‐Gordon chains, using only local measurements. Translating this framework to critical engineering applications, we reconstruct the orbital dynamics of the Tiangong and International Space Stations from public data, revealing nearly identical governing laws despite their distinct architectures. Furthermore, from wind‐tunnel experiments on a scaled suspension bridge, EvLOWN extracts the precise equations of motion capturing complex vortex‐induced vibrations. These results establish EvLOWN as a powerful tool for the data‐driven discovery of governing laws in complex systems where weak nonlinearities play a crucial yet subtle role.

## Graphical abstract

Weak nonlinearities critically shape the long term behavior of oscillatory systems but are difficult to identify from data. A data‐driven framework is introduced to infer governing equations of weakly nonlinear oscillators from sparse and noisy observations. The method is validated on benchmark models and real engineering systems, revealing subtle nonlinear dynamics across scales.

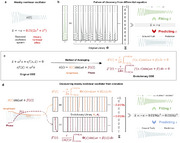

## 1 Introduction

Oscillatory behavior, characterized by rhythmic fluctuations around equilibrium, is a hallmark of complex engineered systems. While idealized harmonic oscillators offer foundational insight, subtle deviations introduced by weak nonlinearities are increasingly recognized as critical to system functionality and stability. These weakly nonlinear oscillators (WNOs) [1], arising from ubiquitous factors such as small perturbations [2], anharmonic potentials [3, 4], and weak coupling effects [5], govern dynamics across scales – from macroscopic orbital motion [6] to microscopic quantum processes [7]. Despite their subtlety, these nonlinearities can lead to pronounced effects, including amplitude‐dependent frequency shifts, modulation instabilities, and nonlinear energy transfer [8, 9, 10]. Such effects are not confined to any single domain but arise universally across physical and engineered systems, where even weak nonlinearities can accumulate and shape long‐term dynamics in profound ways. In atomic lattices and nanostructures, trace anharmonicity modifies phonon dispersion and drastically influences thermal transport [11, 12]. In quantum many‐body systems, weak interactions, beyond simple pairwise models, dictate coherence lifetimes, spectral diffusion, and energy redistribution [13]. In biological oscillators, such as neuronal circuits and circadian clocks, weak nonlinear couplings determine synchronization and phase locking critical to physiological function [14]. Across these diverse domains, overlooking weak nonlinearities as minor corrections risks substantial errors and system failures, undermining both predictive modeling and real‐time control. For example, inaccurate modeling of orbital trajectories contributed to the 2009 satellite collision [15], resulting in billions of dollars in losses and generating space debris that poses a major threat to the Low Earth Orbit environment [16]. In civil engineering, neglecting aeroelastic nonlinearities led to the 1940 collapse of the Tacoma Narrows Bridge due to wind‐induced flutter [17]. In micro‐electromechanical systems, weak nonlinear coupling governs delicate modal interactions essential to functionality [18]. These cases underscore a universal lesson: weak nonlinearities often delineate the boundary between robust operation and catastrophic failure. Addressing them is not merely a modeling challenge but a technological imperative.

To ensure reliable performance, models should capture both dominant dynamics and weak nonlinear effects. While the behaviors of WNOs can be observed experimentally, their underlying dynamics often remain elusive. Traditionally, governing equations for WNOs are derived from first principles, such as conservation laws, or through phenomenological modeling based on expert knowledge. However, these approaches can fall short in real‐world applications, where simplified physics or pre‐assumed functional forms may introduce bias and limit predictive accuracy. The increasing availability of high‐quality observational data now enables a paradigm shift: from assumption‐driven modeling to data‐driven discovery. In recent years, significant progress has been made in data‐driven inference of governing equations, broadly characterized by two complementary methodological paradigms. One line of work focuses on symbolic discovery, which seeks to recover closed‐form representations of physical laws directly from data [19, 20]. A parallel paradigm is sparse regression–based discovery, which aims to identify parsimonious dynamical models within a predefined library of candidate functions [21, 22]. Initially developed for ordinary differential equations and later extended to partial differential equations [23, 24], these approaches together established the foundations of modern equation discovery. Importantly, advances in machine learning theory have enhanced the robustness of these methods to sparse or noisy data [25, 26, 27, 28], making them increasingly viable for real‐world scientific discovery. More broadly, they have catalyzed the emergence of scientific machine learning as a framework for uncovering interpretable dynamical laws from data across disciplines, including cyber‐physical systems [29], fluid dynamics [30, 31], biological, and chemical networks [32, 33] and complex networks [34, 35].

**FIGURE 1:**
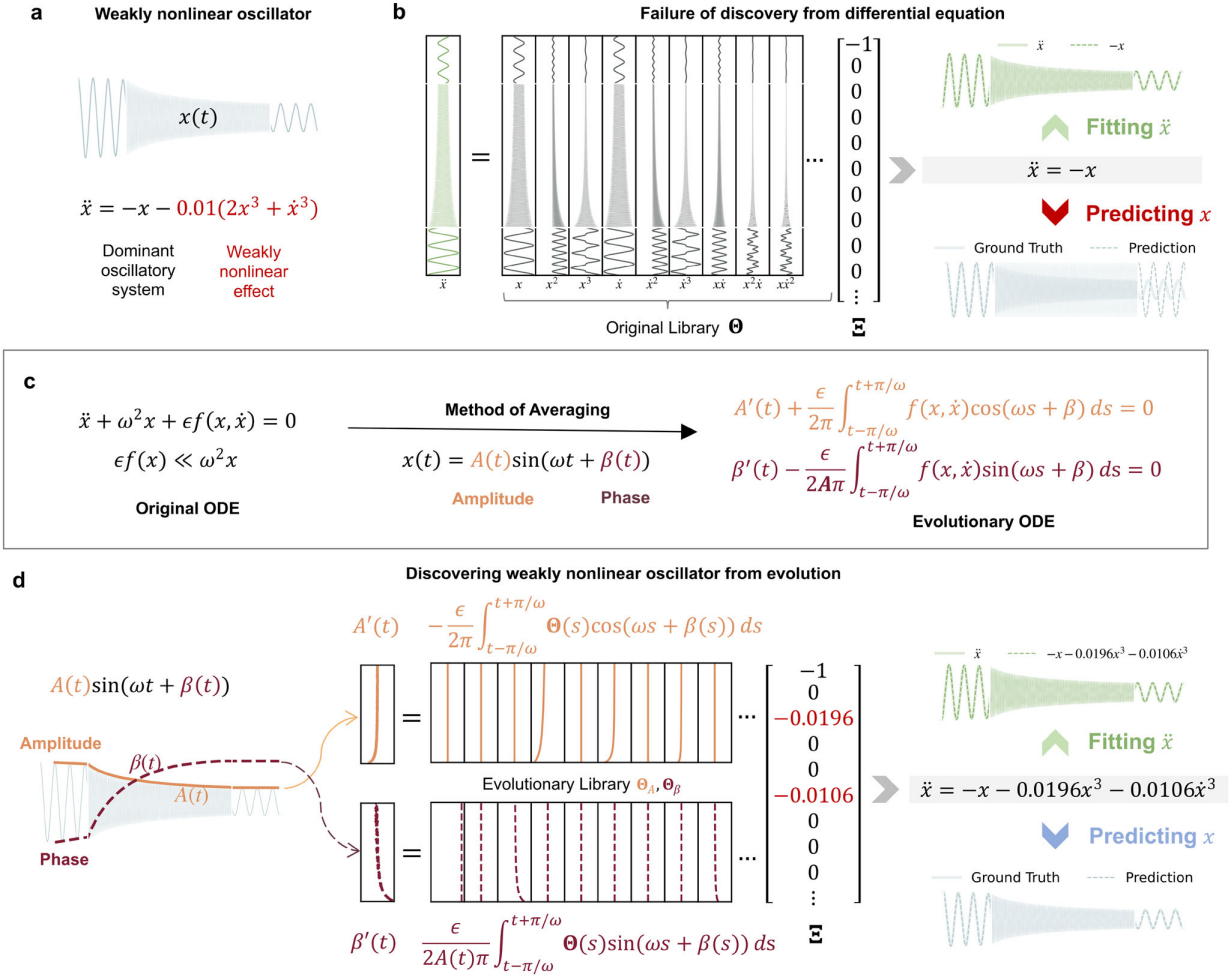
Learning WNOs and the core idea of EvLOWN. (a) Schematic of a WNO and a sample time series. (b) Comparison of instantaneous fitting in traditional data‐driven models of displacement, velocity, and acceleration. With the fitted observed acceleration (e.g., $\ddot{x}=-x$) at a given time t, traditional methods neglect weak nonlinear terms such as $x^{3}$ and ${\dot{x}}^{3}$ whose coefficients are significantly smaller. Hence, the resulting model $\ddot{x}+x=0$ fails to capture the long‐term evolution of x(t). (c) Averaging theory relates weak nonlinear effects to the slow evolution of amplitude A(t) and phase β(t), separating fast and slow dynamics. (d) EvLOWN leverages this formulation by learning governing equations for the evolution variables from a reformulated library. It successfully identifies weak nonlinear terms despite their small magnitude, enabling both accurate reconstruction of $\ddot{x}$ and reliable long‐term prediction of system behavior x(t).

However, existing methods largely overlook the challenges posed by weak nonlinearities in real systems. Most data‐driven approaches focus on extracting concise and dominant governing equations, often discarding terms deemed “insignificant”. This presents a critical limitation when modeling WNOs, where the very terms essential to capturing system behavior may be mistakenly excluded (Figure 1b) – even when clean and complete data are available. The core difficulty lies in the fact that weakly nonlinear terms, by nature, appear subtle in magnitude and are easily filtered out by standard model selection techniques. Compounding this issue, in many oscillator experiments only the displacement x(t) is directly measured, while the conjugate velocity $\dot{x}\left(t\right)$ is formally reconstructible from x(t), numerical differentiation (or weak‐form variants) amplifies measurement noise by powers of $\Delta t^{-1}$, which overwhelms the weakly nonlinear effects.

To address this gap, we pose the central question: Given displacement‐only observations of a complex engineered system, how to learn the governing equations of a weakly nonlinear oscillator (WNO) that capture both dominant oscillatory behavior and weakly nonlinear effects? To this end, we introduce EvLOWN (Evolutionary Learning Oscillator with Weak Nonlinearity), a data‐driven framework for discovering WNO dynamics. EvLOWN reformulates the original system of ODEs into an evolutionary‐variable representation via averaging theory, naturally separating terms by their order of magnitude.

We validate EvLOWN across four representative classes of WNOs—damped harmonic oscillators, limit‐cycle oscillators, systems with anharmonic potentials, and weakly coupled oscillators—covering key physical regimes. Comparative benchmarks show that EvLOWN outperforms four state‐of‐the‐art methods in accurately reconstructing governing equations under varying conditions of term disparity and noise, demonstrating superior robustness and precision. Pushing this further, we demonstrate that from the severely limited data of a single oscillator's local activity, EvLOWN successfully infers the global dynamics of entire Fermi‐Pasta‐Ulam and Klein‐Gordon chains, accurately identifying their subtle nonlinear potentials.

We further apply EvLOWN to two real‐world engineered systems of practical importance: (1) Satellite orbital dynamics of the China Tiangong Space Station (CSS) and the International Space Station (ISS). Using only multi‐day trajectory data, EvLOWN recovers the governing equations with high fidelity; the inferred models yield predictions in close agreement with ground‐truth trajectories. Notably, independent reconstructions for CSS and ISS reveal strikingly similar dynamical laws, highlighting EvLOWN's ability to uncover universal principles in WNOs. (2) Vortex‐induced vibrations (VIV) in bridge aerodynamics. Using wind tunnel data from a 1:20 scale sectional model of a long‐span bridge, EvLOWN accurately extracts governing equations for both free and vortex‐induced vibrations. In particular, it reveals previously overlooked mechanical nonlinearities present even at zero wind velocity, challenging conventional assumptions in VIV modeling.

## 2 Results

### 2.1 Core Idea of the EvLOWN Approach

The dynamics of weakly nonlinear oscillators (WNOs) are generally governed by second‐order ordinary differential equations (ODEs)

(1) $${\ddot{x}}_{i}\left(t\right)+\omega_{i}^{2}x_{i}\left(t\right)+\varepsilon_{i}f_{i}\left(x\left(t\right),\dot{x}\left(t\right)\right)=0\;\text{for}\;i=1,\text{…},d$$

where $\left(x\left(t\right),\dot{x}\left(t\right)\right)$ denotes the state of the WNO at time t, $x\left(t\right)={\left(x_{1}\left(t\right),\text{…},x_{d}\left(t\right)\right)}^{⊤}\in R^{d}$ may represent, for example, displacements in mechanical systems, current in electrical circuits, or positions in celestial mechanics, and $\dot{x}\left(t\right)={\left({\dot{x}}_{1}\left(t\right),\text{…},{\dot{x}}_{d}\left(t\right)\right)}^{⊤}$ is its time derivative; $\omega_{i}\in R$ denotes the natural frequency of the ith component; $f_{i}\left(x,\dot{x}\right)$ captures the weakly nonlinear effects; and $\varepsilon_{i}\ll 1$ is a small parameter quantifying the strength of nonlinearity. Although the nonlinear term $\varepsilon_{i}f_{i}\left(x,\dot{x}\right)$ is typically much smaller in magnitude than the dominant linear term $\omega_{i}^{2}x_{i}$, it plays a crucial role in shaping system stability, synchronization, and long‐term behaviors [5, 36].

In the absence of prior knowledge about the functional form of the nonlinear terms $f=(f_{1},\text{…},f_{d})$, a natural scheme is to adopt symbolic regression [19] or SINDy‐like [21] ideas, constructing a library $\Theta =(\theta_{1},\text{…},\theta_{m})$ comprising a wide range of possible basic terms $\theta_{m}\left(y\right)$ of the full state vector $y=[x,\dot{x}]$, including polynomials, exponentials, and other nonlinearities. While this library cannot span the infinite‐dimensional model space, one can, in principle, include as many plausible terms as needed, although doing so would increases the difficulty, particularly due to the non‐orthogonality and potential redundancy among candidate terms [34].

The equation discovery problem can thus be framed as selecting an optimal subset of terms from Θ that best reproduces the observed system behavior and most closely matches the ground‐truth ODE. Traditionally, this is done by optimizing a sparse coefficient vector $\Xi_{i}=\left(\xi_{i1},\text{…},\xi_{im}\right)$ for each dimension i, typically by solving ${\dot{y}}_{i}\left(t\right)=\Theta \left(t\right)\Xi_{i}$. However, in the case of WNOs, the linear term $\omega_{i}^{2}x_{i}$ dominates the dynamics. As a result, existing data‐driven methods – which often rely on matching instantaneous values of acceleration, velocity, and displacement – tend to overlook the weakly nonlinear term because of its seemingly negligible magnitude. For instance, given the WNO described in Figure 1a, a model identified as ${\ddot{x}}_{i}+\omega_{i}^{2}x_{i}=0$ may reproduce short‐time data with reasonable accuracy, but when integrated over time, it produces entirely incorrect long‐term predictions for the evolution of x (Figure 1b).

The core idea of our approach is to overcome this limitation by learning the evolution of amplitude and phase, rather than directly learning the state variables x. Leveraging averaging theory [37], also known as the Krylov‐Bogoliubov‐Mitropolsky method [38], we approximate a WNO as $x_{i}\left(t\right)=A_{i}\left(t\right)\sin\left(\omega_{i}t+\beta_{i}\left(t\right)\right)$ when the nonlinear effect is weak (i.e., ε is small). In this representation, the amplitude $A_{i}\left(t\right)$ and phase $\beta_{i}\left(t\right)$ evolve much more slowly over time compared to the oscillatory term, changing little over a single period $2\pi /\omega_{i}$. This separation of timescales allows us to average the system's dynamics over one oscillation cycle (Figure 1c), yielding explicit relationships between the weak nonlinearities and the time evolution of the amplitude $A_{i}\left(t\right)$ and phase $\beta_{i}\left(t\right)$:

(2) $$\begin{array}{c} \begin{array}{cc} A_{i}^{\prime }\left(t\right) & =\frac{A_{i}\left(t+\pi /\omega_{i}\right)-A_{i}\left(t-\pi /\omega_{i}\right)}{2\pi /\omega_{i}}\\ & =-\frac{\varepsilon_{i}}{2\pi }\int_{t-\pi /\omega_{i}}^{t+\pi /\omega_{i}}f_{i}\left(x\left(s\right),\dot{x}\left(s\right)\right)\cos\left(\omega_{i}s+\beta_{i}\left(s\right)\right)ds\\ \beta_{i}^{\prime }\left(t\right) & =\frac{\beta_{i}\left(t+\pi /\omega_{i}\right)-\beta_{i}\left(t-\pi /\omega_{i}\right)}{2\pi /\omega_{i}}\\ & =\frac{\varepsilon_{i}}{2A_{i}\left(t\right)\pi }\int_{t-\pi /\omega_{i}}^{t+\pi /\omega_{i}}f_{i}\left(x\left(s\right),\dot{x}\left(s\right)\right)\sin\left(\omega_{i}s+\beta_{i}\left(s\right)\right)ds \end{array} \end{array}$$

where ${\left(\cdot \right)}^{\prime }$ denotes the rate of change over one period. To identify the first‐order ODEs governing the evolution of amplitude and phase, we integrate the basis terms in the original library over a single period, which quantifies their contributions to the averaged dynamics. This transformation results in two new libraries: $\Theta_{A}$ for amplitude evolution and $\Theta_{\beta }$ for phase evolution. The discovery task now becomes identifying a sparse subset of basis terms from these libraries that best approximate $A_{i}^{\prime }\left(t\right)$ and $\beta_{i}^{\prime }\left(t\right)$ (refer to Figure 1d and Method Section for details). Critically, this averaging process naturally separates terms by order of magnitude, allowing weak nonlinear contributions to be isolated from dominant dynamics. An additional advantage of our approach is that it requires only displacement‐only observations, particularly useful in real‐world scenarios where certain measurements, such as velocity, are difficult to obtain and must be approximated via finite‐difference or weak formalism.

**FIGURE 2:**
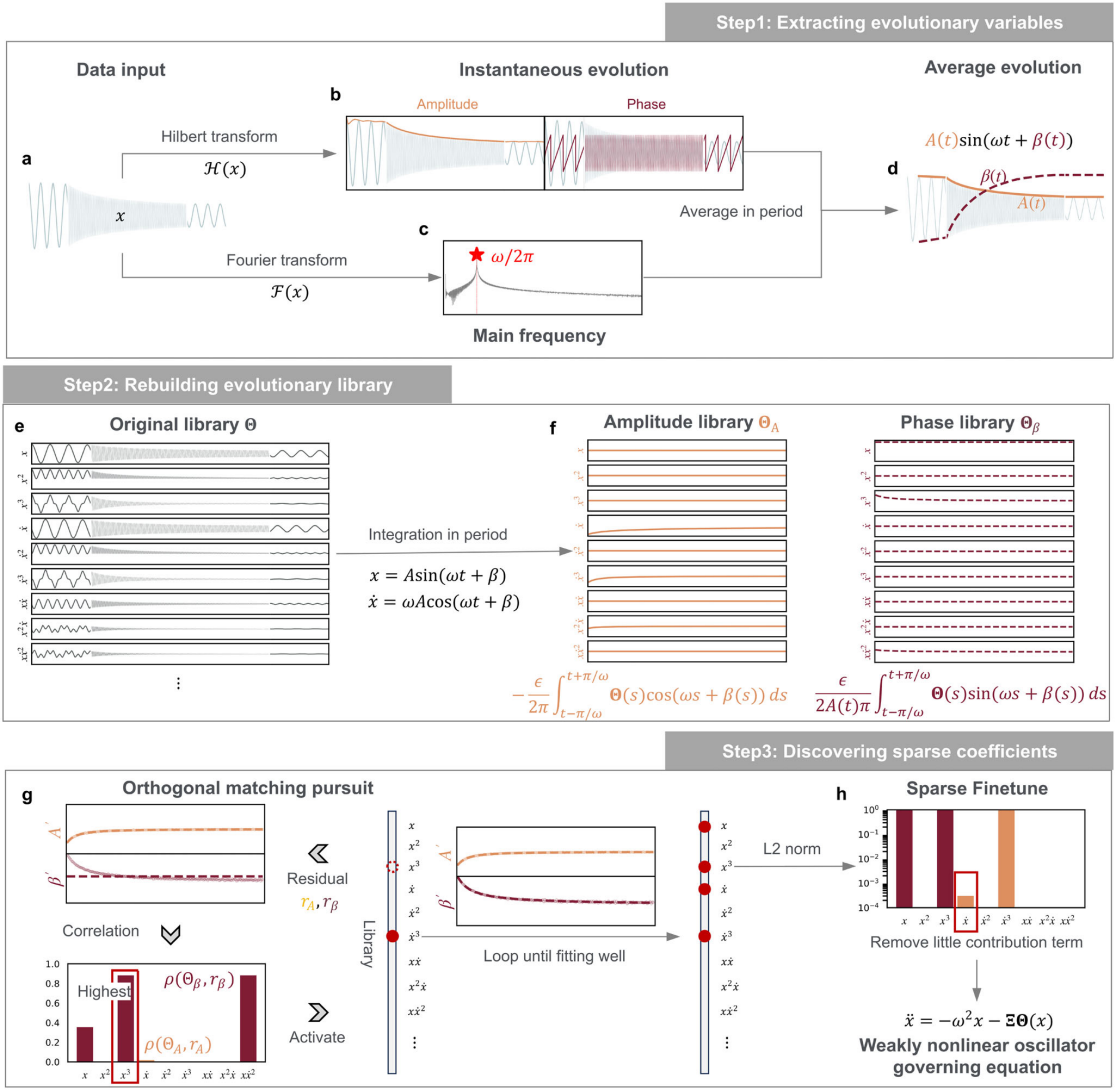
Pipeline of EvLOWN approach: (a–d), Step 1) Extracting evolutionary variables: amplitude and phase are extracted by Hilbert and Fourier transform and averaged in period; (e–f), Step 2) Rebuilding evolutionary library: Libraries for evolution variables are rebuilt integrating over one period; (g–h), Step 3) Discovering sparse coefficients: Two‐stage sparse discovery in evolution space, including orthogonal matching pursuit and sparse finetune.

EvLOWN approach involves three steps: Extracting evolutionary variables, Rebuilding evolutionary library, and Discovering sparse coefficients (Figure 2). In step 1, we extract the oscillating frequencies $\omega_{i}$ and instantaneous evolution by using the Fourier transform $F(x_{i})$ and the Hilbert transform $H(x_{i})$ respectively, and then calculate the average evolution value within the period. Based on the evolution, we can rebuild the evolutionary libraries by integrating the basis functions from the original library in each period. In Step3, we discover the sparse coefficients $\Xi_{i}$, which can satisfy $A_{i}^{\prime }\left(t\right)=\Theta_{A}\left(t\right)\Xi_{i}$ and $\beta_{i}^{\prime }\left(t\right)=\Theta_{\beta }\left(t\right)\Xi_{i}$ at the same time. To discover Ξ, we propose a two‐stage sparse regression method so that the algorithm can find a set of sparse coefficients that simultaneously satisfy two ordinary differential equations. The orthogonal matching pursuit method [39] is first employed to select basis functions by measuring the correlation coefficients between the evolutionary variables and the contributions of the basis functions. The function with the highest correlation coefficient is chosen iteratively until the evolutionary variables are adequately fitted or the improvement in fitting accuracy becomes negligible. After selecting the most relevant functions, we perform a fine‐tuning step with a cut‐off threshold to eliminate functions with little contribution. The final sets of basis functions and their coefficients $\Xi_{i}$ compose $f_{i}$, leading to the inferred dynamics of the WNO.

### 2.2 Numerical Validations and Comparison Experiments

#### 2.2.1 Numerical Validations

**FIGURE 3:**
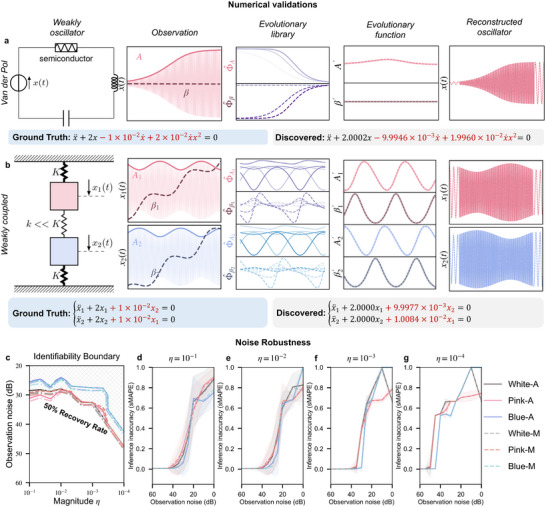
Numerical validation and Noise robustness. (a–b) Application of EvLOWN to two canonical weakly nonlinear oscillator systems: the Van der Pol oscillator and weakly coupled oscillators. In both cases, the weak nonlinear effects are two orders of magnitude smaller than the dominant linear stiffness terms. EvLOWN accurately recovers the full governing equations, including both dominant and weak components, by learning the slow evolution of amplitude and phase. (c–g) Noise‐robustness validation under six different conditions, consisting of two noise types, namely additive (A) and multiplicative (M) Gaussian observational noise, and three power spectral characteristics, namely white, blue, and pink spectra. (c) Identifiability boundaries at different noise levels and nonlinearity magnitudes. A boundary is defined as the parameter region in which the success rate of equation identification exceeds 50% over 100 independent runs. Solid and dash‐dot curves jointly indicate the boundary, and the dash‐dot line denotes the interior of the identifiable region. (d–g) Relative coefficient identification errors under different noise levels and nonlinearity magnitudes.

To evaluate the effectiveness of the EvLOWN approach, it is first applied to infer governing equations for a variety of WNO systems with known ground truth, including the Van der Pol oscillator (Figure 3a), a system of two degrees of freedom (DOF) weakly coupled oscillators (Figure 3b), and both harmonic and Duffing oscillators (Figure S2). In all cases, the weakly nonlinear coefficients are two orders of magnitude smaller than the dominant frequency‐related terms. Data are generated by numerically integrating the ground‐truth equations (Table S2), and only displacement‐only observations x are provided as input to EvLOWN. The results (Figure 3a,b) demonstrate accurate reconstruction of the governing equations and highlight the method's ability to uncover weak nonlinearities. The advantageous capacity of EvLOWN to exploit the slow evolution of amplitude and phase by transforming the original library of candidate terms via the averaging method, which captures the contribution of each basis term to the dynamics of the evolutionary variables. A sparse regression is then employed to select the optimal subset of terms that best matches the observed evolutionary behavior. To further assess the role of weak nonlinearities, both qualitative and quantitative comparisons are performed between the inferred models with and without nonlinear terms and the ground truth (see also Figure S3). These analyses confirm that, although weak nonlinearities are subtle, omitting them during model identification leads to substantial deviations in long‐term dynamic predictions.

#### 2.2.2 Noise Robustness

Due to the minor contribution to differential equations, the weakly nonlinear terms are inherently obscured by observational noise. We further experimentally assess the robustness of our approach under varying levels of observational noise and different magnitudes of weakly nonlinear terms. To systematically evaluate noise robustness, we consider both additive and multiplicative Gaussian observational noise, where the noise processes are generated with three different power spectral characteristics, namely white, blue, and pink spectra, and the overall noise intensity is quantified using the signal‐to‐noise ratio(refer to Section S3.1 for simulation details). Specifically, we assess EvLOWN's robustness in identifying the governing equations of the Van der Pol oscillator system without any denoising pre‐process. As shown in Figure 3c–g, our method exhibits strong robustness to observational noise. Figure 3c–g report the identification boundaries and the relative coefficient estimation errors across different noise levels and weak‐nonlinearity magnitudes η. The boundary is defined as the parameter region in which more than fifty percent of one hundred independent trials successfully recover the correct model. To quantify the difference between the true equation and inferred equation, we introduce symmetric mean absolute percentage error (sMAPE)[22], as detailed in Section S3.3. Our approach can tolerate observational noise of approximately 30 dB and is able to successfully identify weak nonlinear terms that are three to four orders of magnitude smaller than the dominant linear component. The performance remains largely consistent across all six noise conditions considered.

#### 2.2.3 Comparison with Previous Methods

**FIGURE 4:**
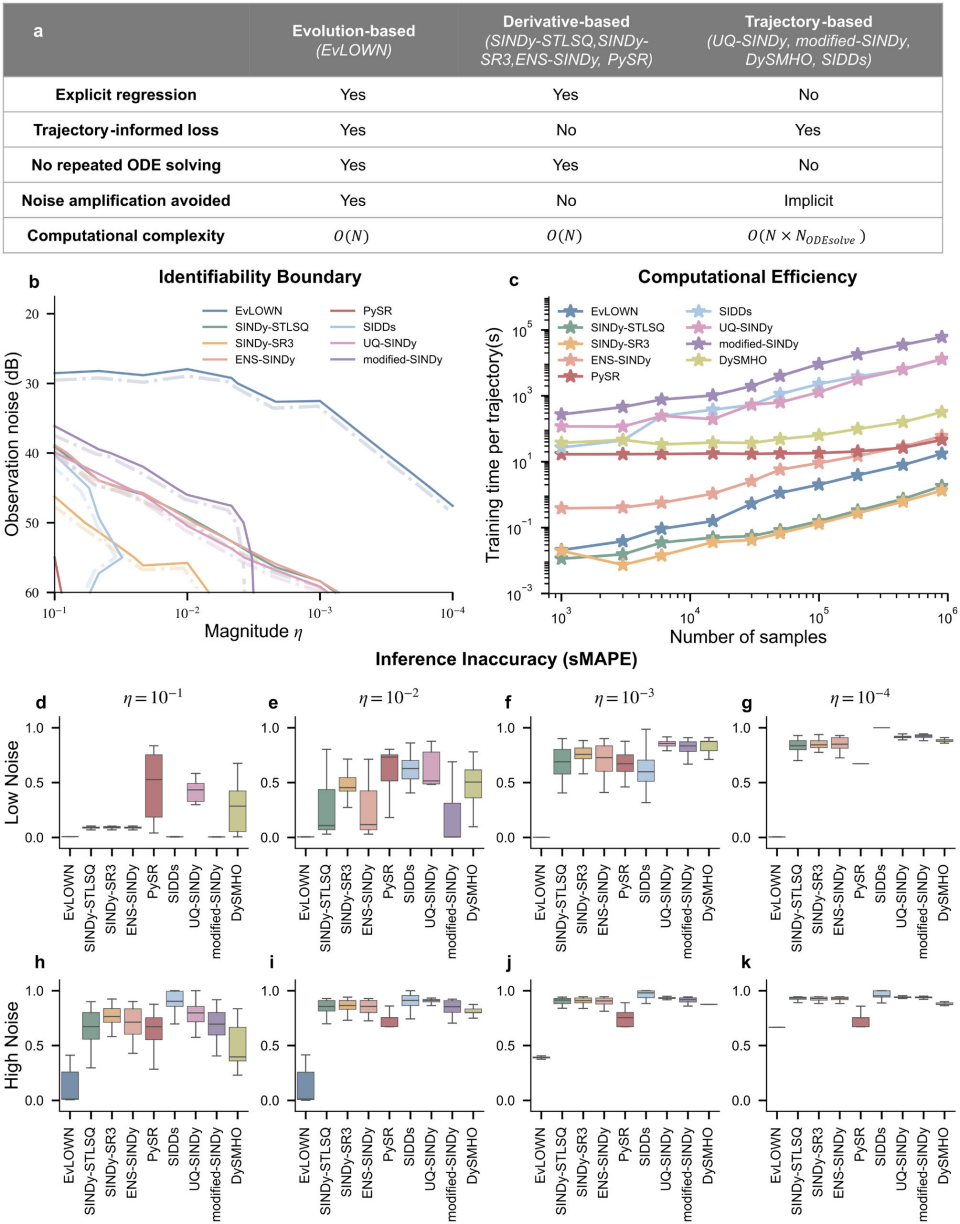
Performance comparison of various models on weakly nonlinear benchmark. (a) Conceptual comparison of representative identification approaches. (b) Identifiability boundaries under different noise levels and nonlinearity magnitudes. A boundary is defined as the region in which the success rate of equation identification exceeds 50% over 100 independent runs. The combination of solid and dashed lines indicates the boundary, and the dashed segment denotes the interior of the identifiable region (Method *DySMHO* does not achieve the 50% succuss ratio in benchmark); (c) the computation time of each algorithm as a function of the input sequence length; (d–g) the relative coefficient identification errors under low observational noise of 55 dB; (h–k) the relative coefficient identification errors under high observational noise of 30 dB.

To underscore the significance of our EvLOWN method in inferring the perturbative nonlinear systems, we conduct comparisons between EvLOWN and representative equation‐discovery approaches, including SINDy‐STLSQ [21], SINDy‐SR3 [40], ENS‐SINDy [41], Symbolic Regression by PySR [42], UQ‐SINDy [26], modified‐SINDy [43], DySMHO [44], and SIDDs [45]. The methods can be broadly grouped into two categories according to the form of their optimization objectives. Derivative‐based methods construct an objective at the level of instantaneous differential equations, whereas trajectory‐based methods formulate the objective using full or partial system trajectories. Figure 4a lists key methodological characteristics. In derivative‐based methods, the learning objective depends only on locally estimated time derivatives. Trajectory information is not explicitly incorporated. Numerical differentiation inevitably amplifies observational noise, while the weak nonlinear terms contribute only weakly to the derivative equation. As a consequence, the weak nonlinear effects are easily submerged by noise and approximation error, which makes reliable identification extremely difficult. In contrast, trajectory‐based methods adopt optimization objectives that directly encode information from system trajectories. In principle, the slow accumulation of weak nonlinear effects along trajectories can therefore be exploited for identification. However, in practice these methods lack closed‐form coefficient updates. The model parameters must be determined through nonlinear gradient‐based optimization frameworks such as Bayesian inference or automatic differentiation platforms. This creates a very large search space, makes convergence to the true model structure difficult, and requires repeated numerical solution of the ODE system during optimization. The resulting computational cost is typically very high. EvLOWN avoids this dilemma. By using the averaging method, it establishes an explicit mathematical connection between the slow evolution of weakly nonlinear systems and the underlying weak nonlinear terms. In this way, trajectory information is incorporated at the level of slow dynamics, while the final identification step remains an explicit regression problem. As a result, EvLOWN retains the advantages of closed‐form coefficient estimation, eliminates the need for large‐scale nonlinear search and repeated ODE simulations, and at the same time significantly enhances the identifiability of weak nonlinear effects.

To substantiate the conceptual analysis, we design a weak‐nonlinearity benchmark using the Van der Pol oscillator, in which we independently vary both the magnitude of the weak nonlinear terms and the level of observational noise. For each parameter combination, we evaluate identifiability(Figure 4b; Figure S7), coefficient‐recovery accuracy(Figure 4d‐k), and computational cost(Figure 4c). The identifiability boundary is determined statistically from 100 independent runs, and we regard a parameter setting as identifiable when the success rate exceeds 50%. The results show that EvLOWN recovers the governing equations reliably across the widest range of weak‐nonlinearity magnitudes and noise levels. It correctly identifies weak nonlinear terms that are several orders of magnitude smaller than the dominant linear contribution while remaining robust to observational noise of about 30 dB. The improved success rate arises from the fact that EvLOWN incorporates trajectory information through slow‐evolution dynamics, which enhances the visibility of weak nonlinear effects, while still retaining an explicit regression formulation. As a consequence, EvLOWN avoids large‐scale nonlinear search and repeated ODE simulations. Compared with trajectory‐based approaches, it achieves comparable or higher accuracy with a computational efficiency advantage of approximately $10^{3}$ to $10^{4}$. Derivative‐based approaches, in contrast, deteriorate as noise increases because numerical differentiation amplifies observation noise and the weak nonlinear contributions to the differential equations are small. Overall, EvLOWN maintains high identification success rates and low coefficient errors with significantly reduced computation time.

### 2.3 Learning Oscillator Chains From Limited Simulated Data

To illustrate EvLOWN's ability to infer governing laws in complex many‐body systems, we further apply it to the oscillator chain, one of the most fundamental prototypical models for studying collective dynamics in physics. Oscillator chains serve as simplified representations of diverse physical systems, including lattice vibrations in crystals [46], nonlinear responses in engineered metamaterials [47], and the dynamics of biomolecular chains such as DNA [48]. Weak nonlinearities are ubiquitous in practical oscillator chains, arising either from the on‐site potentials at each individual site or from the interaction bonds that couple nearest neighbors. Although small in coefficient magnitude, such weak nonlinearities can, through mode coupling, frequency renormalization, and slow intermodal energy transfer, cumulatively reshape the chain—s collective dynamical properties over long times.

**FIGURE 5:**
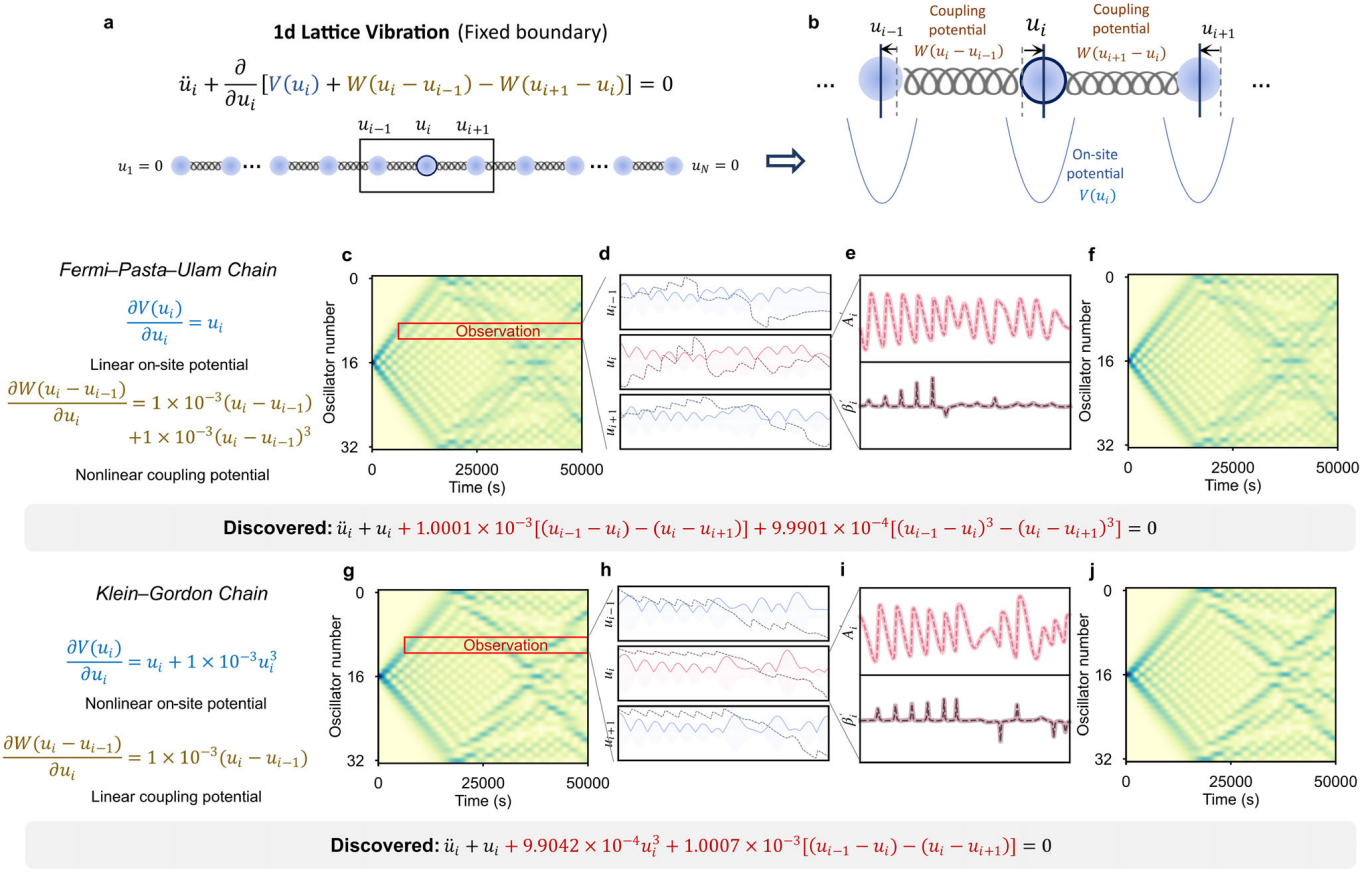
Learning nonlinear oscillator chains from limited data. EvLOWN infers the governing equations of the *Fermi–Pasta–Ulam* and *Klein–Gordon* chains from a single oscillator—s trajectory and its neighbors: (a) 1‐D fixed oscillator chain. (b) Each oscillator i are effected by on‐site potential $V(u_{i})$ and coupling potential with neighbors $W(u_{i}-u_{i-1})$ and $W(u_{i+1}-u_{i})$. (c–f) *Fermi‐Pasta‐Ulam Chain* which has the linear on‐site potential and nonlinear coupling potential. (g–j) *Klein‐Gordon Chain* which has the nonlinear on‐site potential and linear coupling potential. From left to right is simulation data (g), limited observational signal, here is the 10th oscillator and its neighbors (h), slow‐varying evolutions (i), and predictive dynamics from inferred equations (j).

Specifically, we focus on the 1D oscillator chain with fixed boundary conditions(Figure 5a), in which the dynamics of oscillator i is governed by both its intrinsic on‐site potential V and the interaction potentials W arising from couplings with its nearest neighbors(Figure 5b). This example involves two classical chain models: the Fermi–Pasta–Ulam (FPU) chain [49], where nonlinearity arises from couplings, and the Klein–Gordon (KG) chain [50], where nonlinearity originates from the on‐site potentials. In both oscillator chain models, the weak nonlinear effects are approximately three orders of magnitude smaller than the dominant linear interactions. Here, an initial displacement is applied to the middle node of the chain, and the resulting wave propagates slowly along it. Owing to their different weakly nonlinear potentials, the FPU and KG chains exhibit slightly different behaviors, as illustrated in Figure 5c,g, which present heatmaps of the oscillation amplitudes of each node over time.

The data set is generated by numerically integrating the ground‐truth equations (Figure 5), while EvLOWN is trained solely on the activities of a single node $u_{i}\left(t\right)$ together with its nearest neighbors $u_{i-1}\left(t\right),u_{i+1}\left(t\right)$, thus learning the global dynamics from only local observations(Figure 5d,h). For node i, the nonlinearities stem from both the on‐site potential $V(u_{i})$ and the coupling potentials $W(u_{i}-u_{i-1})$ and $W(u_{i+1}-u_{i})$. Based on these two sources, we construct separate libraries to represent the on‐site and coupling contributions. These libraries are subsequently employed to approximate the slowly varying evolutionary variables of $u_{i}$ (Figure 5e,i) and to accurately infer the governing equations while capturing subtle nonlinear effects. We analyse the resulting amplitude evolutions using numerical simulations, and we show good agreement between ground truth(Figure 5c,g) and inferred equations(Figure 5f,j).

### 2.4 Learning Orbital Dynamics of Space Station From Measured Data

**FIGURE 6:**
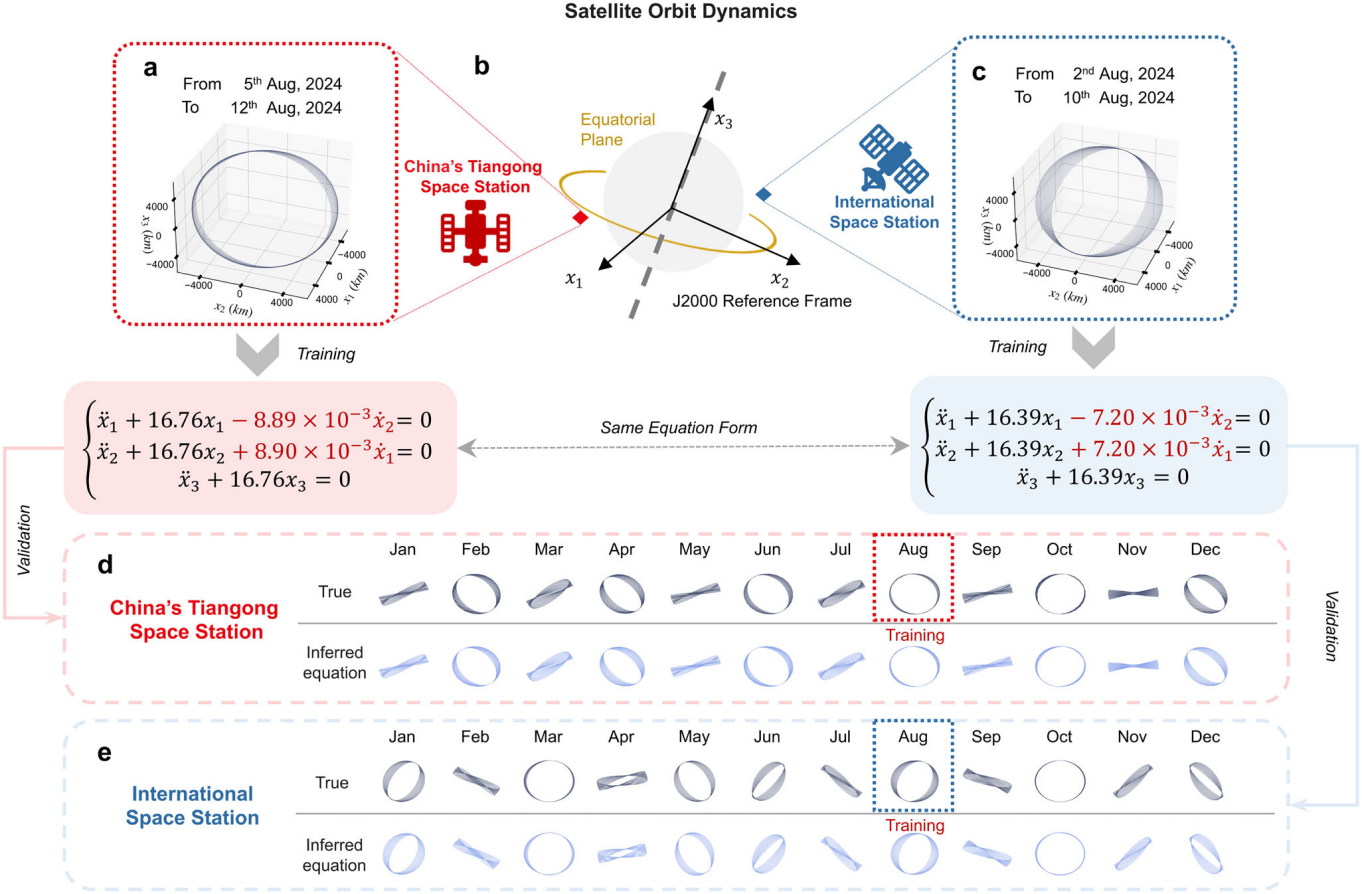
Learning governing equations of satellite orbital dynamics from observational data. (a) Recorded trajectory of China—s Tiangong Space Station (CSS) from August 5 to August 12, 2024. (b) Orbital motion in low Earth orbit, with $x_{1}$, $x_{2}$, and $x_{3}$ denoting position components in the J2000 reference frame (aligned with Earth—s rotation axis and equatorial plane). (c) Recorded trajectory of the International Space Station (ISS) from August 2 to August 10, 2024. (d–e) Comparison of ground‐truth orbital trajectories (from OEM data) and those simulated from the inferred equations for CSS and ISS across 12 distinct time windows.

We apply the EvLOWN approach to two representative real‐world cases, the first of which concerns orbital mechanics, a branch of astrodynamics that studies the motion of objects in outer space [51]. The classical two‐body problem, whose solution yields the unperturbed orbit, serves as the foundational model in celestial mechanics. However, real‐world orbital motion is influenced by various perturbations, including gravitational forces from third bodies, atmospheric drag, and irregularities in gravitational fields caused by oblate or non‐spherical celestial bodies [52]. Given the inherent challenges in accurately modeling multiple disturbance sources, directly inferring celestial dynamics from observed orbital data becomes significantly valuable. Because perturbative forces are typically much weaker than the dominant gravitational attraction of a primary body, the resulting motion can be regarded as a generalized form of WNO. To illustrate the broader applicability of EvLOWN, we infer the underlying orbital dynamics of two operational space stations, the China Tiangong Space Station (CSS) and the International Space Station (ISS), using observed trajectory data (Figure 6a–c).

The EvLOWN approach infers the governing equations of space station motion using only publicly available Orbital Ephemeris Message (OEMP) data collected over a few days (Figure 6a,c). The ISS and CSS datasets are trained independently, yet EvLOWN identifies two sets of governing equations with highly similar forms. To validate the inferred models, 24 trajectories from time periods distinct from the training data, spanning January to December, are used for prediction (see Tables S5 and S7). The close agreement between the predicted and true trajectories (Figure 6d,e) confirms that EvLOWN accurately captures the underlying orbital dynamics. More importantly, the identified governing equations are physically interpretable and recover key orbital perturbation mechanisms, including atmospheric drag in low‐Earth orbit and the J2 perturbation, both of which appear as velocity‐dependent terms in the theoretical model [53].

Beyond validation, this framework offers a transformative solution to the growing risks posed by space debris and collisions with inactive satellites. By autonomously learning high‐fidelity orbital dynamics from limited, real‐time observational data (e.g., 5‐day ephemeris segments), EvLOWN enables predictive, data‐driven collision avoidance even for derelict, tumbling satellites with unknown perturbation histories. Traditional physics‐based methods, which rely on precise initial conditions and comprehensive environmental models, often fail for aging satellites with degraded hardware or undocumented maneuvers. In contrast, the data‐driven EvLOWN approach continuously updates its models to capture subtle but crucial nonlinear perturbations, such as anomalies in solar radiation pressure or residual atmospheric drag, that drive long‐term orbital evolution. As congestion in low‐Earth orbit intensifies, with over 30,000 tracked debris objects threatening critical space infrastructure, EvLOWN provides a scalable, physics‐informed AI tool that enhances orbital safety to support the 1.5 trillion global space economy.

### 2.5 Learning Dynamics of Vortex‐Induced Vibration From Experimental Data

The second representative application concerns vortex‐induced vibration (VIV), a phenomenon commonly observed in engineering systems. VIV arises, for example, in external flows past bluff bodies when the frequency of vortex shedding approaches the natural frequency of the structure [8]. Despite extensive effort, a complete analytical description of VIV remains elusive, and current predictive methods remain rudimentary due to the rich and complex dynamics that are not yet fully understood [54]. Because the mass of an individual vortex and the resulting force it exerts are both small relative to the structural mass and stiffness, VIV dynamics can be effectively characterized as a WNO.

VIV plays a critical role in various engineering domains, including wind, ocean, and aerospace engineering. Over the past several decades, VIV events have frequently been reported on long‐span bridges worldwide, often causing economic disruptions and traffic problems [55]. A notable recent example occurred on May 5, 2020, when the Humen Bridge, which spans the Pearl River connecting Guangzhou and Dongguan, experienced large‐amplitude VIV for the first time in its 23‐year operational history. The incident led to a 10‐day suspension of both vehicular and maritime traffic, significantly impacting the regional economy [56]. Motivated by such real‐world occurrences, the EvLOWN approach is applied to observed VIV response data from a long‐span suspension bridge, aiming to identify the governing equations that characterize the underlying VIV dynamics, including structural motion and vortex shedding interactions.

**FIGURE 7:**
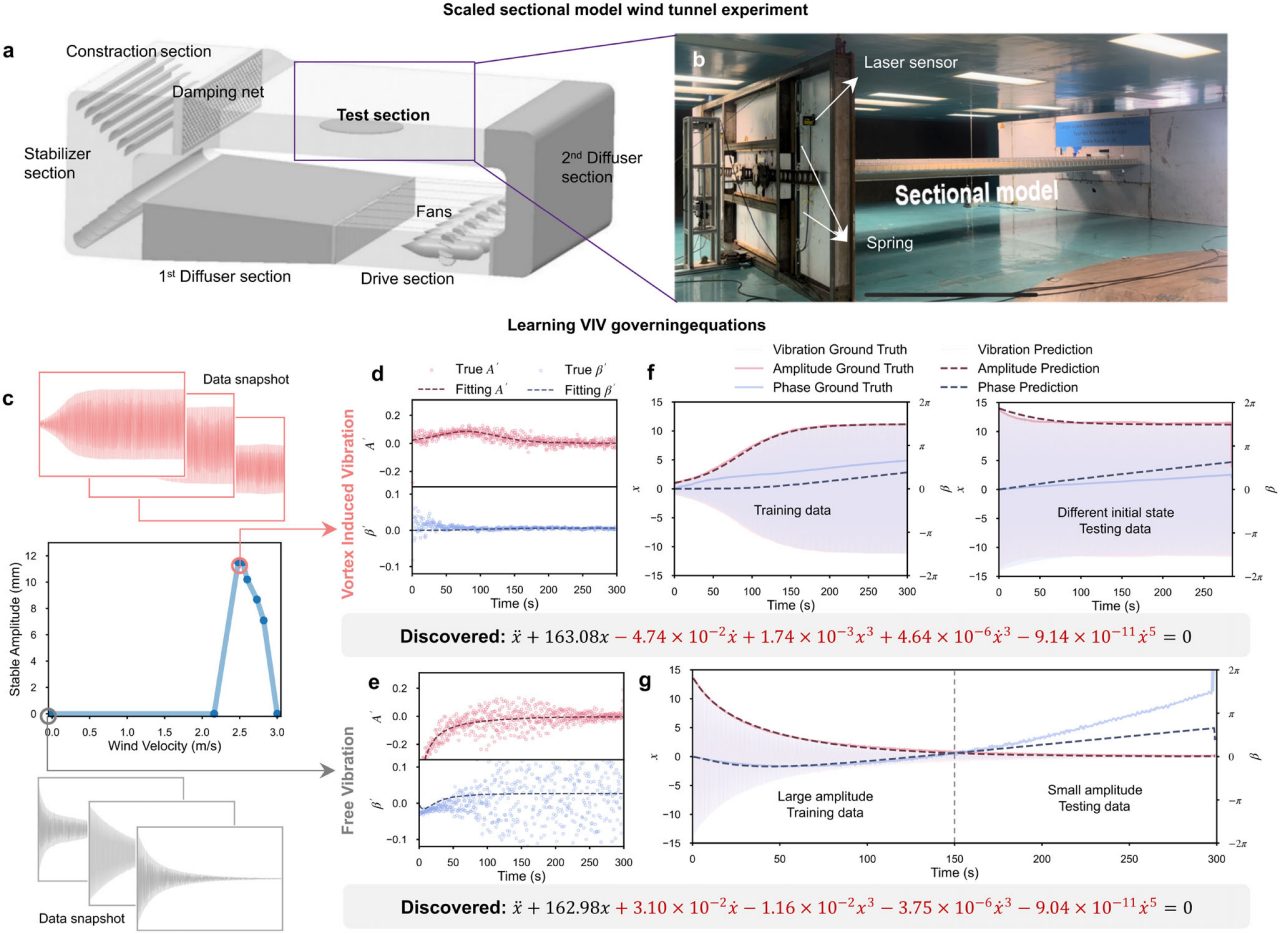
Learning vortex‐induced vibrations (VIV) from experimental data. (a–b) Wind tunnel experiments on a 1:20 scale model of the Xihoumen bridge deck: (a) TJ‐3 boundary wind tunnel facility, (b) photo of the wind tunnel setup, (c–g) Learning governing equations from experimental vibration data: (c) steady‐state amplitudes and sample data snapshots at different wind speeds; (d,f) inferred evolutionary ODEs capturing free vibration (at zero wind) and vortex‐induced vibration (at wind velocity 2.54 m s^−1^); (e,g) validation of the learned models on datasets with varied initial conditions.

To acquire the necessary VIV data, we conduct wind tunnel experiments using a 1:20 scale sectional model of a suspension bridge in the TJ‐3 wind tunnel at Tongji University (Figure 7a,b). The prototype of the model is the Xihoumen Bridge (Figure S9a– c), which has experienced hundreds of VIV events in the past decade [57]. The experimental setup employs ad‐hoc control devices (Figure S9f– g) to position the model at predefined initial states before release. The model is suspended using eight linear springs to replicate the structural stiffness of the actual bridge. The scaling relationships between the model and the prototype are detailed in Tables S10 and S11, while the spring stiffness values are obtained from tensile tests (Figure S10). Vertical oscillation responses x(t), representing VIV behavior under various wind speeds and initial conditions, are recorded using laser displacement sensors at a sampling rate of 300 Hz. The measured stable oscillation amplitudes as a function of wind velocity are shown in Figure 7c. The results clearly exhibit VIV phenomena within the wind speed range of 2.45 to 2.73 m s^−1^.

Using the experimental data, EvLOWN successfully infers concise and effective governing equations that capture varying magnitudes of weak nonlinear effects (Figure 7d‐g; Figures S11 and S12). In the absence of ground‐truth equations for real systems, we evaluate the validity of the inferred models by testing their generalizability to previously unseen initial conditions. Specifically, for the vortex‐induced vibration (VIV) scenarios, the model is trained on data from small initial displacements and tested on data involving larger displacements (Figure 7f). Conversely, for free vibration cases, training is performed on large‐amplitude oscillations, while testing involves small‐amplitude responses (Figure 7g). Notably, the inferred equations contain several terms with small coefficients that are typically neglected in standard modeling approaches. These weak nonlinear terms capture both the nonlinear vortex‐induced aerodynamic forcing and the intrinsic geometric nonlinearity of the bridge structure, the latter of which is rarely accounted for in conventional models [58]. Although these effects are small in magnitude, they play a crucial role in shaping the long‐term oscillatory behavior, and their explicit identification highlights the ability of EvLOWN to reveal subtle but physically meaningful mechanisms underlying weakly nonlinear dynamics. To assess the functional relevance of these small‐amplitude terms, we conducted ablation studies (Figures S13 and S14). In each study, individual terms are systematically removed from the inferred equations, and the resulting impact on system performance is evaluated. These analyses reveal that although the exclusion of small‐coefficient terms minimally affects the fit of the differential equation itself, it significantly alters the long‐term evolution of the system state x(t). This again highlights the subtle yet critical role such weak nonlinear terms play in accurately capturing the full dynamical behavior. Our results also reveal that nonlinear effects are present not only in VIV cases involving vortex shedding but also in free vibration scenarios at zero wind velocity, an aspect often overlooked in conventional VIV studies. Importantly, all governing equations are inferred autonomously from observed data without imposing any prior assumptions about their structure, demonstrating the capacity of EvLOWN to uncover hidden dynamics of complex systems with fluid‐structure interactions.

## 3 Conclusion

Data‐driven inference of dynamical systems is a cornerstone of autonomous scientific discovery. While prior studies have predominantly focused on strongly nonlinear dynamics – successfully capturing dominant effects for concise system representations—they often overlook weak nonlinearities. Yet, these subtle effects can be crucial for the long‐term performance and safety of complex engineered systems. In contrast, this work introduces EvLOWN, a novel data‐driven framework designed to infer the governing equations of weakly nonlinear oscillators (WNOs).

EvLOWN harnesses averaging theory to assess the contributions of candidate terms to slow‐varying dynamics, enabling it to distinguish between dominant and subtle effects. This allows it to remain robust under conditions of high noise and large disparities in term magnitude. Through extensive numerical benchmarks, we demonstrate EvLOWN's superior accuracy and robustness in identifying governing equations for canonical WNO systems, outperforming existing methods, particularly in the presence of observational noise.

Importantly, we apply EvLOWN to two representative classes of real‐world engineered systems: (1) the orbital dynamics of the Tiangong and International Space Stations, and (2) vortex‐induced vibrations in suspension bridges. In both cases, EvLOWN discovers physically interpretable equations that reveal previously overlooked weak nonlinearities. For the orbital systems, the inferred models recover key perturbation mechanisms, including atmospheric drag and the J2 gravitational effect, while for the bridge vibrations, they capture the nonlinear aerodynamic forcing acting on the structure. In particular, the method relies only on displacement measurements and does not require finite difference estimation of unobserved quantities such as the velocity $\dot{x}\left(t\right)$, which significantly enhances its practicality for real‐world applications.

While EvLOWN demonstrates strong performance and provides new insights into real weakly nonlinear oscillators, several important avenues remain open for future research. First, although the present work focuses on systems dominated by a single oscillatory mode with weak nonlinear corrections, many real‐world systems involve mixtures of strong and weak nonlinearities or multiple interacting frequencies. Extending EvLOWN to such regimes, where subtle, weak effects are embedded within more complex or even chaotic dynamics, represents an important direction for future research and a necessary step toward broader applicability to higher‐dimensional and strongly nonlinear systems. Second, the current formulation assumes deterministic dynamics. Generalizing the framework to stochastic differential equations would broaden its applicability to noisy natural environments. Finally, although we have verified EvLOWN on two representative engineered systems, the same mechanism of weak nonlinear modulation arises in biological and physiological oscillators, and we plan to explore applications such as multiscale biochemical rhythms and coupled cellular oscillations in future work.

In summary, EvLOWN addresses a key blind spot in data‐driven modeling: the discovery of subtle yet essential nonlinearities in oscillatory dynamics. It offers both a practical tool for modeling real‐world systems and a conceptual advance in understanding how small effects can shape complex behavior.

## 4 Methods

### 4.1 Method of Averaging

The method of averaging was a mathematical method for the approximate analysis of oscillating processes in nonlinear dynamics [37]. The method was based on the averaging principle when the exact differential equation of dynamics (Equation (1)) was replaced by its averaged version (Equation (2)). Here, we take the 1D problem as an example for detailed derivation:

When the ε=0, the solution of Equation (1) can be written as:

(3) $$\begin{array}{cc} x & =A\sin(\omega t+\beta )=A\sin(\phi ) \end{array}$$

(4) $$\begin{array}{cc} \dot{x} & =A\omega \cos(\omega t+\beta )=A\omega \cos(\phi ) \end{array}$$

where A and β are constants. When ε≠0, the solution can still be expressed in the form (Equation (3) and (4)) provided that A and β are considered to be functions of t rather than constants. To determine the equations describing A(t) and β(t), we differentials Equations (3) and (4) with respect to t and obtain:

(5) $$\begin{array}{cc} \dot{x} & =\omega A\cos\left(\phi \right)-\dot{A}\sin\left(\phi \right)+A\dot{\beta }\cos\left(\phi \right) \end{array}$$

(6) $$\begin{array}{cc} \ddot{x} & =-\omega^{2}A\sin\left(\phi \right)+\omega \dot{A}\cos\left(\phi \right)-\omega A\dot{\beta }\sin\left(\phi \right) \end{array}$$

Comparing Equations (4) and (5), we have Equation (7). Then substituting for x(Equation (3)) and $\ddot{x}$(Equation (6)) in Equation (1), we obtain Equation (8).

(7) $$\begin{array}{cc} \dot{A}\sin\left(\phi \right)+A\dot{\beta }\cos\left(\phi \right) & =0 \end{array}$$

(8) $$\begin{array}{cc} \dot{A}\cos\left(\phi \right)-A\dot{\beta }\sin\left(\phi \right) & =-\frac{\varepsilon }{\omega }f\left(x\right) \end{array}$$

Solving Equations (7) and (8) for $\dot{A}$ and $\dot{\beta }$, we obtain:

(9) $$\begin{array}{cc} \dot{A} & =-\frac{\varepsilon }{\omega }\cos\left(\phi \right)f\left(x\right)\\ \dot{\beta } & =\frac{\varepsilon }{\omega A}\sin\left(\phi \right)f\left(x\right) \end{array}$$

For small ε, $\dot{A,}$ and $\dot{\beta }$ were small; hence A and β hardly change during the period of oscillation 2π/ω of sin(ϕ) and cos(ϕ). This enables us to average out the variables in ϕ in Equation (9). Averaging these equations over the period 2π/ω and considering A,β, $\dot{A}$, and $\dot{\beta }$ to be constants while performing the averaging, we obtain the Equation (2) describing the slow variations of evolutions.

### 4.2 Evolutionary Library

The final goal of this work was to learn both the structure and corresponding coefficients of weakly nonlinear dynamics $f=\left(f_{1},\text{…},f_{d}\right)$. We construct a comprehensive library $\Theta \left(t\right)\in R^{m}$ to represent weakly nonlinear dynamics f. To separate the weakly nonlinear dynamics $f_{i}\left(x\right)$ from dominant oscillating frequency and infer the compact forms that best match ${\ddot{x}}_{i}\left(t\right)+\omega_{i}^{2}x_{i}\left(t\right)+\varepsilon_{i}f_{i}\left(x\left(t\right)\right)=0$, method of averaging recast it to two first‐order ODEs of evolutionary variables $A_{i}^{\prime }\left(t\right)$ and $\beta_{i}^{\prime }\left(t\right)$:

(10) $$\begin{array}{cc} A_{i}^{\prime }\left(t\right) & =\Theta_{A}\left(t\right)\Xi_{i}^{T}=-\frac{1}{2\pi }\left(\int_{t-\pi /\omega_{i}}^{t+\pi /\omega_{i}}\Theta \left(s\right)\cos\left(\omega_{i}s+\beta_{i}\left(s\right)\right)ds\right)\Xi_{i}^{T} \end{array}$$

(11) $$\begin{array}{cc} \beta_{i}^{\prime }\left(t\right) & =\Theta_{\beta }\left(t\right)\Xi_{i}^{T}=\frac{1}{2A_{i}\left(t\right)\pi }\left(\int_{t-\pi /\omega_{i}}^{t+\pi /\omega_{i}}\Theta \left(s\right)\sin\left(\omega_{i}s+\beta_{i}\left(s\right)\right)ds\right)\Xi_{i}^{T} \end{array}$$

where ${\left(\cdot \right)}^{\prime }$ is the operator on taking derivative with respect to period, $\Theta_{A}$ and $\Theta_{\beta }$ encode the patterns of evolutions; $\Xi_{i}\in R^{m}$ is the coefficients of library Θ in $i^{th}$ dimension; $A_{i}\left(t\right),\beta_{i}\left(t\right)$ were the amplitude and phase of $i^{th}$ dimension at time t respectively; $\omega_{i}$ is the circular frequency of oscillation in $i^{th}$ dimension.

To obtain the evolutionary libraries $\Theta_{A}$ and $\Theta_{\beta }$ from original library Θ, the evolution variables $A\left(t\right)=\left(A_{1}\left(t\right),\text{…},A_{d}\left(t\right)\right),\beta \left(t\right)=\left(\beta_{1}\left(t\right),\text{…},\beta_{d}\left(t\right)\right)$ and oscillating frequency $\omega =\left(\omega_{1},\text{…},\omega_{d}\right)$ were required. Given a input data $x\left(t\right)=(x_{1}\left(t\right),\text{…},x_{d}\left(t\right))$, the oscillating frequency $\omega_{i}$ could be calculated by Fourier transform F (Equation (12)). Then the Hilbert transform H was used to compute the analytic signal $x_{i}^{a}\left(t\right)=x_{i}\left(t\right)+iH\left(x_{i}\left(t\right)\right)$. The evolutionary variables $A_{i}\left(t\right)$ and $\beta_{i}\left(t\right)$ of time t could be considered as the averaging value of instantaneous evolutionary variables in one period (Equations (13) and (14)).

(12) $$\begin{array}{cc} \omega_{i} & =\underset{\omega_{i}}{\arg\max}F\left(x_{i}\left(t\right)\right)=\underset{\omega_{i}}{\arg\max}\int_{-\infty }^{+\infty }x_{i}\left(t\right)e^{-i\omega_{i}t}dt \end{array}$$

(13) $$\begin{array}{cc} A_{i}\left(t\right) & =\frac{\int_{t-\pi /\omega_{i}}^{t+\pi /\omega_{i}}\left|x_{i}^{a}\left(s\right)\right|ds}{2\pi /\omega_{i}} \end{array}$$

(14) $$\begin{array}{cc} \beta_{i}\left(t\right) & =\frac{\int_{t-\pi /\omega_{i}}^{t+\pi /\omega_{i}}\mathrm{Arg}\left(x_{i}^{a}\left(s\right)\right)ds}{2\pi /\omega_{i}} \end{array}$$

### 4.3 Sparse Regression

To infer the sparse coefficients vector Ξ that best matches both Equation (10) and Equation (11), we solve the optimization formula Equation (15), which was the generalized regression problem with $L_{1}$ regularization. Due to the non‐differentiable points in the objective function, the optimization formula did not have a closed‐form solution for the global minimum [59]. Several optimization strategies, such as coordinate descent [60] and least angle regression [61], can be applied to address this issue. However, after integrating over one period, the evolutionary libraries disrupt the orthogonality between different basis functions (Supporting Information), making these strategies less effective. Hence, we proposed a two‐stage procedure consisting of orthogonal matching pursuit and sparse fine‐tune.

(15) $$\begin{array}{c} \underset{\Xi_{i}}{\arg\min}\int_{0}^{T}\left(\left|\right|A_{i}^{\prime }\left(t\right)-\Theta_{A}\left(t\right)\Xi_{i}{\left|\right|}_{2}^{2}+\left|\right|\beta_{i}^{\prime }\left(t\right)-\Theta_{\beta }\left(t\right)\Xi_{i}{\left|\right|}_{2}^{2}\right)dt+\lambda ||\Xi_{i}{\left|\right|}_{1} \end{array}$$

where $\left|\right|\cdot {\left|\right|}_{2}^{2}$ is the least squares term; $\left|\right|\cdot {\left|\right|}_{1}$ is the $L_{1}$ norm of the coefficient vector Ξ, which is the sum of absolute values of the coefficients; λ is the regularization parameter that controls the strength of the penalty.

Stage one: The orthogonal matching pursuit (OMP)[39] was a greedy algorithm that selected the most relevant basis functions to the true dynamics of WNO. OMP builds a sparse solution to a given evolutionary variables $A_{i}^{\prime }\left(t\right)$ and $\beta_{i}^{\prime }\left(t\right)$ by iteratively building up an approximation; the evolutionary variables were approximated as a linear combination of a few basis functions of evolutionary libraries $\Theta_{A}$ and $\Theta_{\beta }$, where the active set to be used was built one by one in a greedy fashion. The orthogonal matching pursuit algorithm is shown in Algorithm 1. For each dimension i of the system, the active set L was initially set to the empty set. At each iteration, the correlation coefficient $\rho_{ij}$ between each basis function j and residual $r_{A},r_{\beta }$ is computed and the basis function that best correlated with the current residual was added to the active set. Then the coefficient vector $\Xi_{i}$ was computed by the least squared method (Equation (17)) and residuals were updated by Equations (18) and (19). The loop stops when the $L_{2}$ norm of the residual was small enough or the increment of the residual is small enough.

(16) $$\begin{array}{cc} \rho_{ij} & =\frac{{\hat{\Theta }}_{Aij}r_{A}}{|{\hat{\Theta }}_{Aij}{|}_{2}{\left|r_{A}\right|}_{2}}+\frac{{\hat{\Theta }}_{\beta ij}r_{\beta }}{|{\hat{\Theta }}_{\beta ij}{|}_{2}{\left|r_{\beta }\right|}_{2}} \end{array}$$

(17) $$\begin{array}{cc} \Xi_{i} & ={\left({\hat{\Theta }}_{Ai}^{T}{\hat{\Theta }}_{Ai}+{\hat{\Theta }}_{\beta i}^{T}{\hat{\Theta }}_{\beta i}\right)}^{-1}\left({\hat{\Theta }}_{Ai}^{T}A_{i}^{\prime }+{\hat{\Theta }}_{\beta i}^{T}\beta_{i}^{\prime }\right) \end{array}$$

(18) $$\begin{array}{cc} r_{A} & =A_{i}^{\prime }-{\hat{\Theta }}_{Ai}\Xi_{i} \end{array}$$

(19) $$\begin{array}{cc} r_{\beta } & =\beta_{i}^{\prime }-{\hat{\Theta }}_{\beta i}\Xi_{i} \end{array}$$

where the subscript ${\left(\cdot \right)}_{j}$ represents the jth basis function and subscript ${\left(\cdot \right)}_{i}$ represents the ith dimension of system; ${\hat{\Xi }}_{i}\in R^{m}$ was the coefficient vector during optimization; ${\hat{\Theta }}_{Ai},{\hat{\Theta }}_{\beta i}\in R^{m\times n}$ were the active matrices updated by active set L.

ALGORITHM 1 **Orthogonal matching pursuit**

**Table:** 

1:	**for** i=1,2,…,d **do** ▹ Loop each dimension of system
2:	$r_{A}\leftarrow {\dot{A}}_{i};r_{\beta }\leftarrow {\dot{\beta }}_{i}$ ▹ Initialize residual
3:	L←∅ ▹ Initialize the active set
4:	**for** iteration=1,2,… **do**
5:	Compute the correlation coefficient $\rho_{ij}$ between each basis function $L_{j}$ and residual $r_{A},r_{\beta }$ (Equation (16))
6:	Select the basis function with the highest correlation coefficient and add to L
7:	Update the active matrices ${\hat{\Theta }}_{i}$ based on L and compute the evolutionary matrices $\Theta_{Ai},\Theta_{\beta i}$ by Equations (10) and (11)
8:	Compute the coefficient vector $\Xi_{i}$ by Equation (17) and Update the residual $r_{A},r_{\beta }$ by Equations (18) and (19)
9:	**end for**
10:	**end for**

Stage two: Sparse finetune was used to remove the little contribution terms for a generalized and concise formula of f. After orthogonal matching pursuit, the model space was narrowed down by selecting the most relevant basis functions. The contribution $c_{ij}$ of each basis function j in ith dimensions was calculated by Equation (20) and then normalized to 0–1. These contributions were utilized to fine‐tune the selected basis functions. To do so, we remove the basis functions whose contribution value was lower than the cut‐off threshold.

(20) $$\begin{array}{c} c_{ij}=\int_{0}^{T}\left(\frac{{\left({\hat{\Theta }}_{Aij}\left(t\right)\Xi_{ij}\right)}^{2}}{{\left(A_{i}^{\prime }\left(t\right)\right)}^{2}}+\frac{{\left({\hat{\Theta }}_{\beta ij}\left(t\right)\Xi_{ij}\right)}^{2}}{{\left(\beta_{i}^{\prime }\left(t\right)\right)}^{2}}\right)dt \end{array}$$

### 4.4 Hyper‐Parameters

The hyperparameters of EvLOWN approach are defined in what follows.

1. residual tolerance: In the orthogonal matching pursuit stage, when the residual after adding a basis function was less than this residual tolerance, the loop terminates and moves to the sparse finetune stage.
2. increment of residual tolerance: In the orthogonal matching pursuit stage, when the difference between the residual after adding a basis function and the last loop was less than the increment of residual tolerance, the loop terminates and moves to the sparse finetune stage.
3. weight ratio: In the orthogonal matching pursuit stage, the weight ratio between amplitude and phase in correlation coefficient ρ computation.
4. lowest contribution ratio: In the sparse fine‐tune stage, the basis function whose contribution was lower than the lowest contribution ratio will be removed from the final result.

### 4.5 Inference Uncertainty Analyze

In order to analyze the uncertainty of the inferred equations, we extend EvLOWN with a statistical procedure to quantify the confidence of inferred equations. To estimate the confidence of inferred coefficients from a single dataset, which represents the most common scenario in real‐world applications, we adapt the bootstrap aggregating strategy and incorporate it into EvLOWN. In EvLOWNs framework, two categories of inferred terms are considered: dominant linearity and weak nonlinearity. For dominant linearity, Fourier Transform was applied to block bootstrap samples of the input time series to obtain an ensemble of oscillatory frequencies. The mean oscillatory frequency was then used to construct evolutionary variables (amplitude and phase) and to transform the evolutionary library. For weak nonlinearity, orthogonal matching pursuit was repeatedly performed on index bootstrap samples of the slow‐varying evolutionary variables to generate an ensemble of nonlinear terms. It was worth noting that block bootstrap resamples contiguous segments of the time series to preserve temporal dependence, ensuring that Fourier Transform yields valid oscillatory frequencies for every bootstrap sample.

### 4.6 Statistical Analysis

For Figure 3d–g, all results were obtained from 100 samples, and the mean values and 68.27% confidence intervals(mean±sd) were plotted. For Figure 4c, all results were obtained from ten independent samples, and the mean values were plotted. For Figure 4d–k, all results were obtained from 100 samples, and the median, quartile, and whisker were plotted.

## Author Contributions

Conceptualization: T.M., W.C.; Methodology: T.M.; Experiment: T.M., W.C., L.Z.; Visualization: T.M., W.C., T.T.G.; Funding acquisition: W.C., L.Z.; Results Discussion: T.M., T.T.G. W.C., L.Z., G.Y., A.F. Project administration: L.Z.; Supervision: W.C., L.Z., A.F., G.Y.; Writing – original draft: T.M.; Writing – review & editing: T.M., T.T.G. W.C., L.Z., G.Y., A.F.

## Conflicts of Interest

The authors declare no conflicts of Interest.

## Supporting information


**Supporting File**: advs74325‐sup‐0001‐SuppMat.pdf.

## Data Availability

All data are provided in the main text or the supplementary materials. The codes are available in the public GitHub repository: https://github.com/TengMa25/EvLOWN.git
