# Supplementary material for: Encoding Cumulation to Learn Perturbative Nonlinear Oscillatory Dynamics
Source: Adv Sci (Weinh). 2026 Mar 6;13(25):e19707. doi: 10.1002/advs.202519707 (PMC13137823; doi:10.1002/advs.202519707)
Supplement: Supplementary file 1 — Supporting File: advs74325‐sup‐0001‐SuppMat.pdf. [file ADVS-13-e19707-s001.pdf]

Supplementary Information for "Encoding cumulation to learn perturbative nonlinear oscillatory dynamics"

Teng Ma[1,2], Ting-Ting Gao[3], Wei Cui[1*], Attilio Frangi[2], Gang Yan[4,5*] and Lin Zhao[1,6*]

1. *State Key Lab of Disaster Reduction in Civil Engineering, Tongji University; Shanghai 200092, P. R. China*
2. *Department of Civil and Environmental Engineering, Politecnico di Milano; Milan 20133, Italy*
3. *Network Science Institute, Northeastern University, Boston, MA 02115, USA*
4. *MOE Key Laboratory of Advanced Micro-Structured Materials, and School of Physical Science and Engineering, Tongji University; Shanghai 200092, P. R. China.*
5. *Shanghai Research Institute for Intelligent Autonomous Systems, National Key Laboratory of Autonomous Intelligent Unmanned Systems, MOE Frontiers Science Center for Intelligent Autonomous Systems, and Shanghai Key Laboratory of Intelligent Autonomous Systems, Tongji University; Shanghai 200092, P. R. China*
6. *Key Laboratory of Disaster Prevention and Structural Safety of China Ministry of Education, Guangxi University; Nanning 530004, P. R. China*

Contents

1 Approach **3**
- 1.1 Oscillating frequency estimation . . . 3
- 1.2 Basis functions in the evolutionary library . . . 5

2 Numerical examples **5**

3 Details of Noise robustness **9**
- 3.1 Data Generation . . . 9
- 3.2 Steady state amplitude error . . . 10
- 3.3 Quantification of inference inaccuracy . . . 11

4 Details of Comparison experiments **11**
- 4.1 Description of baseline methods . . . 11
- 4.2 Details of weakly nonlinear benchmark . . . 12
- 4.3 Inference results . . . 13
- 4.4 Discussion about DAHSI . . . 14

5 Satellite orbit dynamics **15**
- 5.1 China's Tiangong Space Station . . . 16
- 5.2 International Space Station . . . 17

6 Vortex-induced vibration case **17**
6.1 Wind tunnel experiment setup 18
6.2 Inferring results for other wind speeds 20
6.3 Influence of weak terms 20

1 Approach

1.1 Oscillating frequency estimation

In EvLOWN, the base frequency ω is estimated from the data using Fourier analysis and serves as a key input for constructing the slow–fast decomposition. Accurate identification of ω is therefore important for separating the fast oscillatory component from the slow amplitude and phase dynamics. However, somewhat counterintuitively, small errors in the estimated frequency do not lead to systematic bias in the recovered weak nonlinear terms. In this section, we show analytically that even if the estimated frequency contains a small perturbation, the resulting error only affects the slow phase variable and is subsequently corrected during the sparse regression stage. We provide a formal derivation to demonstrate why EvLOWN remains robust to small frequency estimation errors.

Consider a weakly nonlinear oscillator

$$\ddot{x} + \omega^2 x + \varepsilon f(x, \dot{x}) = 0, \tag{S1}$$

where $0 < \varepsilon \ll 1$. In EvLOWN, the base frequency is estimated from data. We denote the estimated frequency by

$$\hat{\omega} = \omega + \delta\omega, \tag{S2}$$

where $\delta\omega$ is the estimation error. Since ω is obtained from the dominant spectral peak, we assume

$$\delta\omega = O(\varepsilon), \tag{S3}$$

so that the frequency mismatch enters the slow dynamics at the same order as the weak nonlinearity.

Using $\hat{\omega}$ as the reference frequency, Eq. (S1) can be equivalently rewritten as

$$\ddot{x} + \hat{\omega}^2 x + \varepsilon \hat{f}(x, \dot{x}) = 0, \tag{S4}$$

where the effective forcing term is defined by

$$\hat{f}(x, \dot{x}) = f(x, \dot{x}) + (\omega^2 - \hat{\omega}^2)\, x. \tag{S5}$$

Equivalently, $f(x, \dot{x}) - \hat{f}(x, \dot{x}) = (\hat{\omega}^2 - \omega^2)x$. Therefore, a frequency estimation error introduces an additional weak linear forcing term proportional to $(\omega^2 - \hat{\omega}^2)x$.

Following the averaging procedure used in the main text, we represent the solution as

$$x(t) = \hat{A}(t) \sin\bigl(\hat{\omega} t + \hat{\beta}(t)\bigr),$$

where $\hat{A}(t)$ and $\hat{\beta}(t)$ evolve on the slow time scale. Let $\phi = \hat{\omega} t + \hat{\beta}(t)$. To leading order, we have

$$x = \hat{A} \sin\phi, \qquad \dot{x} = \hat{A}\hat{\omega} \cos\phi.$$

Using the standard averaging formulas (given in the main text) with the effective forcing $\hat{f}$, the slow-flow equations take the form

$$\dot{\hat{A}} = -\frac{1}{2\pi\hat{\omega}} \int_0^{2\pi} \varepsilon \hat{f}(\hat{A}\sin\phi, \hat{A}\hat{\omega}\cos\phi)\cos\phi \, d\phi, \tag{S6}$$

$$\dot{\hat{\beta}} = \frac{1}{2\pi\hat{\omega}\hat{A}} \int_0^{2\pi} \varepsilon \hat{f}(\hat{A}\sin\phi, \hat{A}\hat{\omega}\cos\phi)\sin\phi \, d\phi. \tag{S7}$$

We now examine the contribution of the frequency-error term

$$(\omega^2 - \hat{\omega}^2)x = (\omega^2 - \hat{\omega}^2)\hat{A}\sin\phi$$

in (S6)–(S7).

Amplitude equation. Substituting $(\omega^2 - \hat{\omega}^2)\hat{A}\sin\phi$ into (S6) yields an additional term proportional to

$$-\int_0^{2\pi} \sin\phi\cos\phi \, d\phi = 0.$$

Hence, the frequency estimation error does not affect $\dot{\hat{A}}$ to leading order.

Phase equation. Substituting $(\omega^2 - \hat{\omega}^2)\hat{A}\sin\phi$ into (S7) yields an additional term

$$\frac{1}{2\pi\hat{\omega}\hat{A}} \int_0^{2\pi} (\omega^2 - \hat{\omega}^2)\hat{A}\sin^2\phi \, d\phi = \frac{1}{2\pi\hat{\omega}}(\omega^2 - \hat{\omega}^2) \int_0^{2\pi} \sin^2\phi \, d\phi.$$

Since $\int_0^{2\pi} \sin^2\phi \, d\phi = \pi$, we obtain

$$\dot{\hat{\beta}} = \dot{\beta}_{\text{true}} + \frac{1}{2\hat{\omega}}(\omega^2 - \hat{\omega}^2), \tag{S8}$$

where $\dot{\beta}_{\text{true}}$ denotes the slow phase evolution that would be obtained using the exact frequency.

Using $\hat{\omega} = \omega + \delta\omega$ and $\delta\omega = O(\varepsilon)$, we have

$$\frac{1}{2\hat{\omega}}(\omega^2 - \hat{\omega}^2) = \frac{1}{2\hat{\omega}}(\omega - \hat{\omega})(\omega + \hat{\omega}) \approx -\delta\omega$$

Therefore, to leading order,

$$\dot{\hat{\beta}} \approx \dot{\beta} - \delta\omega, \tag{S9}$$

which shows that a small frequency estimation error introduces only a constant drift in the slow phase, while leaving the amplitude equation unchanged.

Consistency with the signal representation. The same physical trajectory can be written as

$$x(t) = A(t)\sin\big(\omega t + \beta(t)\big) = \hat{A}(t)\sin\big(\hat{\omega}t + \hat{\beta}(t)\big).$$

Substituting $\hat{\omega} = \omega + \delta\omega$ and matching the phases implies

$$\hat{A}(t) = A(t), \qquad \hat{\beta}(t) = \beta(t) - \delta\omega\, t,$$

and hence

$$\dot{\hat{\beta}} = \dot{\beta} - \delta\omega.$$

This is consistent with Eq. (S9) up to the assumed scaling $\delta\omega = O(\varepsilon)$ and the slow-time normalization used in the averaging derivation. In summary, a small frequency estimation error manifests primarily as a drift in the slow phase variable and does not affect the amplitude dynamics nor the identifiability of weak nonlinear terms.

1.2 Basis functions in the evolutionary library

The basis functions in the evolutionary libraries are computed from $\mathbf{\Theta}$ according to:

$$\begin{aligned}\mathbf{\Theta}_A &= \frac{1}{2\pi\boldsymbol{\omega}}\int_0^{2\pi} \mathbf{\Theta}\cos(\boldsymbol{\phi})d\boldsymbol{\phi} \\ \mathbf{\Theta}_\beta &= -\frac{1}{2\boldsymbol{A}\pi\boldsymbol{\omega}}\int_0^{2\pi} \mathbf{\Theta}\sin(\boldsymbol{\phi})d\boldsymbol{\phi}\end{aligned} \tag{S10}$$

where $\mathbf{\Theta}_A$ and $\mathbf{\Theta}_\beta$ encode the patterns of evolutions; $\boldsymbol{A} = (A_1, A_2, ..., A_d)^T$ and $\boldsymbol{\beta} = (\beta_1, \beta_2, ..., \beta_d)^T$ are d-dimensional evolutionary variables; $\boldsymbol{\phi} = (\phi_1, \phi_2, ..., \phi_d)^T = \boldsymbol{\omega}t + \boldsymbol{\beta}$, where $\boldsymbol{\omega} = (\omega_1, \omega_2, ..., \omega_d)$ is the oscillating circular frequencies of each dimension.

Due to this integration step, many basis functions are no-longer independent, making the traditional optimization strategies, such as coordinate descent and least angle regression less effective. To further explain this point, let us consider the example of the oscillator $\ddot{x} + \omega_0^2 x + \epsilon f(x, \dot{x}) = 0$ and the candidate functions $x^2\dot{x}$ and $\dot{x}^3$ for $f(x, \dot{x})$:

$$\begin{aligned}\Theta_A(x^2\dot{x}) &= -\frac{1}{2\pi\omega_0}\int_0^{2\pi}\cos(\phi)(x^2\dot{x})d\phi = -\frac{1}{2\pi\omega_0}\int_0^{2\pi}\cos(\phi)(A\sin(\phi))^2 A\omega_0\cos(\phi)d\phi = \frac{0.25A^3}{2\pi} \\ \Theta_\beta(x^2\dot{x}) &= -\frac{1}{2A\pi\omega_0}\int_0^{2\pi}\sin(\phi)(x^2\dot{x})d\phi = -\frac{1}{2A\pi\omega_0}\int_0^{2\pi}\sin(\phi)(A\sin(\phi))^2 A\omega_0\cos(\phi)d\phi = 0\end{aligned} \tag{S11}$$

$$\begin{aligned}\Theta_A(\dot{x}^3) &= -\frac{1}{2\pi\omega_0}\int_0^{2\pi}\cos(\phi)(\dot{x}^3)d\phi = -\frac{1}{2\pi\omega_0}\int_0^{2\pi}\cos(\phi)(A\omega_0\cos(\phi))^3 d\phi = \frac{0.75\omega_0^2 A^3}{2\pi} \\ \Theta_\beta(\dot{x}^3) &= -\frac{1}{2A\pi\omega_0}\int_0^{2\pi}\sin(\phi)(\dot{x}^3)d\phi = -\frac{1}{2A\pi\omega_0}\int_0^{2\pi}\sin(\phi)(A\omega_0\cos(\phi))^3 d\phi = 0 = 0\end{aligned} \tag{S12}$$

It can be seen that $x^2\dot{x}$ and $\dot{x}^3$ provide the same contributions in terms of evolutionary variables and are therefore equivalent. To further elaborate on this, we simulate a weakly nonlinear oscillator with parameters $\epsilon = -0.01$ and $\omega_0 = 10$ (Supplementary Figure S5) and two different choices for f that yield the very same evolution. It should be noted that this is not restricted to $x^2\dot{x}$ and $\dot{x}^3$, as there are many candidate functions that are totally equivalent for evolutionary variables, even if they originate from independent polynomial basis functions.

2 Numerical examples

In the main text, we demonstrate the effectiveness of EvLOWN in inferring governing equations of four WNOs: Harmonic and Duffing oscillators, respectively. The validations of Harmonic and Duffing

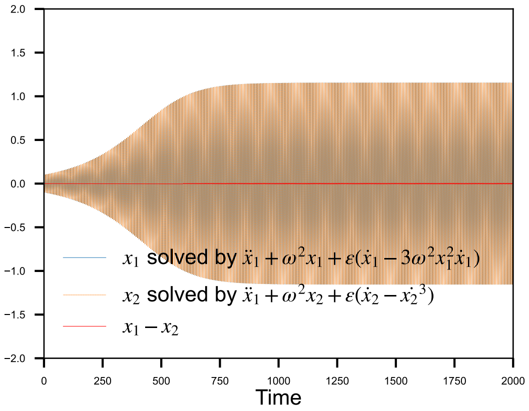

Supplementary Fig. S1: Comparison between two WNOs with equivalent weakly nonlinear effects ($3\omega^2 x^2 \dot{x}$ and $\dot{x}^3$)

ocsillator is shown in Supplementary Fig. S2 The libraries Φ adopted for these WNOs are listed in Supplementary Table $S1$.

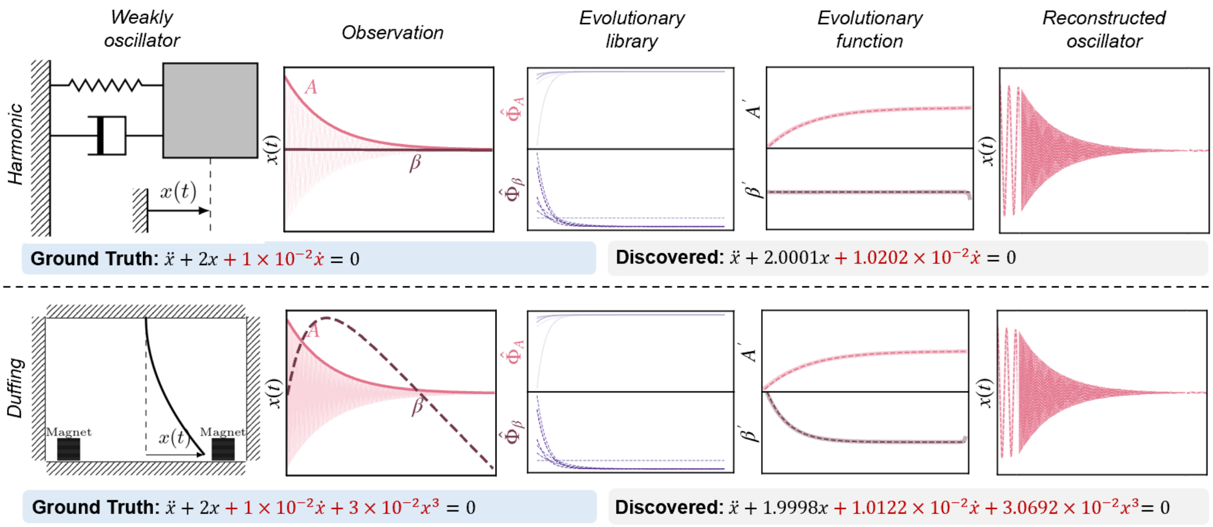

Supplementary Fig. S2: WNO learning applied to two classical examples, including Damped-harmonic and Duffing oscillators. Their weak effects all have two orders of magnitude difference compared with stiffness. Our approach can learn the correct governing equations accurately, including stiffness and weak effects, from amplitude and phase evolution

To ensure the reproducibility of our results, we list all the parameters utilized in Supplementary Table S2. The time span of the simulations is $[0 - T]$ with step-size Δt. The hyper-parameters of EvLOWN are listed in Supplementary Table S3.

Supplementary Figure S3 evaluates the accuracy of the inferred governing equations $\ddot{x} = \hat{F}$ with four representative weakly nonlinear oscillators (WNOs): harmonic, van der Pol, Duffing and weakly coupled systems. For each class, we validate the results using two criteria: (1) Long-term trajectory prediction: Solving $\ddot{x} = \hat{F}(x, \dot{x})$ yields $\hat{x}(t)$ trajectories (orange curves) that closely align with ground truth $x(t)$ (black curves), with sub-percent deviations over 2000 cycles (left panels a, c, e, g). (2) Equation fidelity: Direct comparison between the inferred equation $\hat{F}$ and the true equation F (right panels b, d, f, h). These results collectively demonstrate that EvLOWN not only captures subtle weak nonlinearities, but also

Supplementary Tab. S1: Libraries adopted in the numerical examples

Trajectory	State vector	Library Φ
Harmonic	$[x, \dot{x}]$	$[x, \dot{x}, x^2, x\dot{x}, \dot{x}^2, x^3, x^2\dot{x}, x\dot{x}^2, \dot{x}^3, x^4, x^3\dot{x}, x^2\dot{x}^2, x\dot{x}^3, \dot{x}^4, x^5, x^4\dot{x}, x^3\dot{x}^2, x^2\dot{x}^3, x\dot{x}^4, \dot{x}^5]$
Van der Pol		
Duffing		
Weakly Coupled	$[x_1, \dot{x}_1, x_2, \dot{x}_2]$	$[x_1, \dot{x}_1, x_2, \dot{x}_2, x_1^2, x_2^2, \dot{x}_1^2, \dot{x}_2^2, x_1\dot{x}_1, x_1x_2, x_1\dot{x}_2, \dot{x}_1x_2, \dot{x}_1\dot{x}_2, x_2\dot{x}_2]$

Supplementary Tab. S2: Summary of parameters utilized in the simulations

Dynamics	Dimension	Time history data	Initial conditions $[x_0, \dot{x}_0]$
Harmonic	1	$T = 1000$<br>$\Delta t = 0.05$	$[2, 0]$
Van der Pol	1	$T = 1000$<br>$\Delta t = 0.05$	$[0.1, 0]$
Duffing	1	$T = 1000$<br>$\Delta t = 0.05$	$[2, 0]$
Weakly Coupled	2	$T = 2000$<br>$\Delta t = 0.05$	$[2, 1, 2, 0]$

preserves their cumulative impact on long-term system evolution, a critical advance beyond conventional data-driven approaches that prioritize instantaneous fitting accuracy at the expense of dynamical realism.

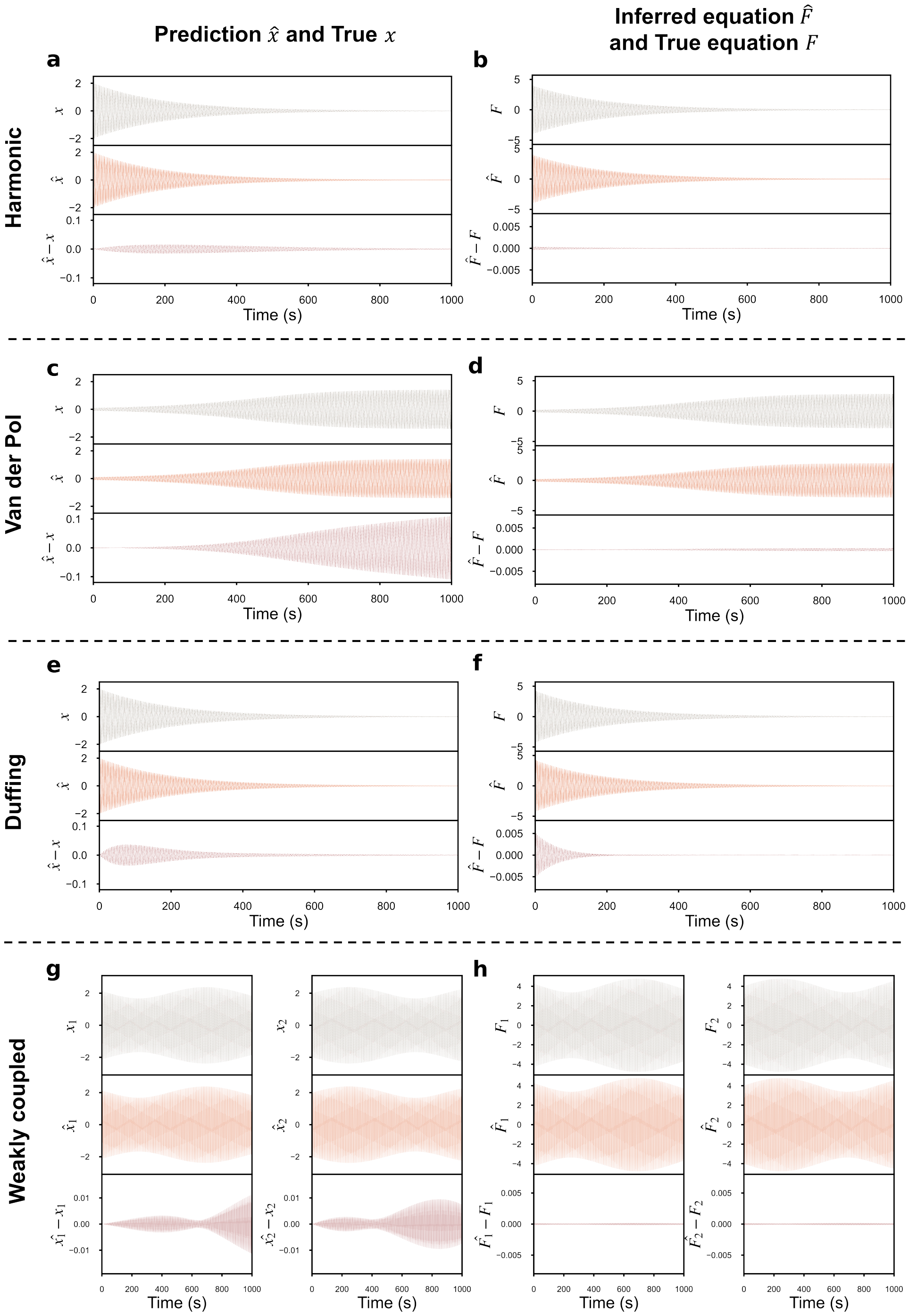

Supplementary Fig. S3: Accuracy assessment of inferred governing equations $\hat{F}$ for four classes of weakly nonlinear oscillators (WNOs): (a, b) harmonic; (c, d) Van der Pol; (e, f) Duffing; (g, h) weakly coupled. Left panels (a,c,e,g): Quantitative trajectory comparisons between ground truth x (black) and predicted solution $\hat{x}$ (orange) by solving inferred equations $\ddot{x} = \hat{F}(x, \dot{x})$. Right panels (b,d,f,h): Fitting comparisons between inferred equation $\hat{F}$ and true equation F.

Supplementary Tab. S3: Hyper-parameters for EvLOWN used in numerical examples

Dynamics	residual tolerance	increment of residual tolerance	lowest contribution ratio	Weight ratio
Harmonic	1×10^{-4}	1×10^{-4}	1×10^{-2}	1
Van der Pol	1×10^{-3}	1×10^{-2}	1×10^{-2}	1
Duffing	1×10^{-4}	1×10^{-4}	1×10^{-1}	1
Weakly coupled	1×10^{-4}	1×10^{-4}	1×10^{-1}	1

3 Details of Noise robustness

3.1 Data Generation

In this section, we validate the robustness of EvLOWN with respect to observational noise and to the magnitude of weak nonlinear terms. We generate synthetic data from the ground-truth weakly nonlinear oscillator and then corrupt the observations using both additive and multiplicative noise processes with different spectral characteristics.

For additive noise, the observed signal is given by

$$\begin{aligned} \ddot{x} + 10x + \eta(2x^2 - 1)\dot{x} &= 0 \\ x^{obs} &= x + aN(t) \end{aligned} \tag{S13}$$

where η is a scale factor of weakly non-linear terms and a is a multiplicative parameter of a white Gaussian noise, with $N(t)$ normal distribution with zero mean and standard deviation.

For multiplicative noise, the observations are constructed as

$$\begin{aligned} \ddot{x} + 10x + \eta(2x^2 - 1)\dot{x} &= 0 \\ x^{obs} &= x + ax(t)N(t) \end{aligned} \tag{S14}$$

The overall noise intensity is quantified by the signal-to-noise ratio (SNR), reported in decibels. Throughout this work, SNR is defined as the ratio between the empirical power of the clean signal and that of the noise term in the corresponding observation model, with larger SNR values indicating weaker observational noise.

To investigate spectral effects, we further consider three types of noise power spectra: white, blue, and pink. These colored noise processes are generated by shaping Gaussian white noise in the frequency domain to match the prescribed spectral slopes. Representative examples and their power spectral densities are shown in Fig. S*, illustrating the differences among the three noise types.

$$
\begin{aligned}
&\mathcal{F}\{N(t)\} = \hat{N}(f), \\
&\mathbb{E}\left[|\hat{N}(f)|^2\right] \propto f^{\alpha}, \\
&\alpha = \begin{cases} 0, & \text{white noise}, \\ 1, & \text{blue noise}, \\ -1, & \text{pink noise}. \end{cases}
\end{aligned} \tag{S15}
$$

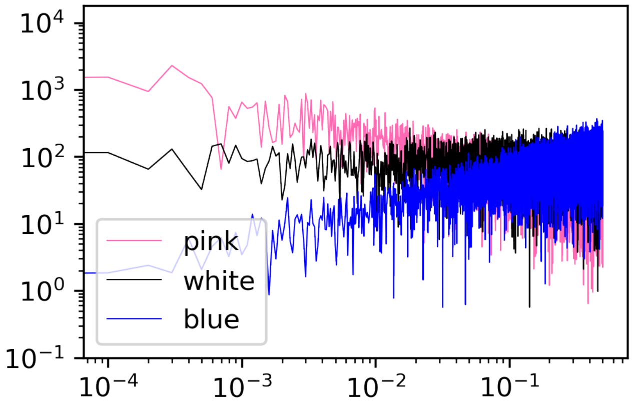

Supplementary Fig. S4: Colored noise example in time domain (from top to bottom: pink noise, white noise and blue noise)

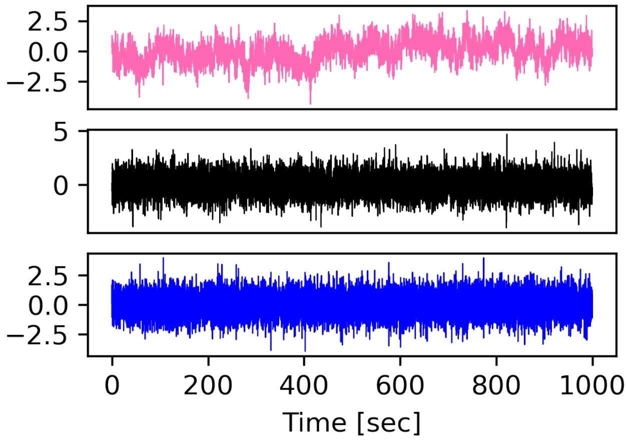

Supplementary Fig. S5: Colored noise example in frequency domain (from top to bottom are pink, white and blue noise respectively)

For each combination of noise type, SNR level, and weak-nonlinearity magnitude, we perform 100 independent trials with different noise realizations and estimate the identification success rate from the resulting statistics.

3.2 Steady state amplitude error

The steady state oscillation amplitude is a key dynamical feature of the Van der Pol oscillator. We use it as a quantitative measure to assess whether the learned system preserves the underlying dynamics. As an example, we analyze the effect of additive Gaussian white noise on the identification results.

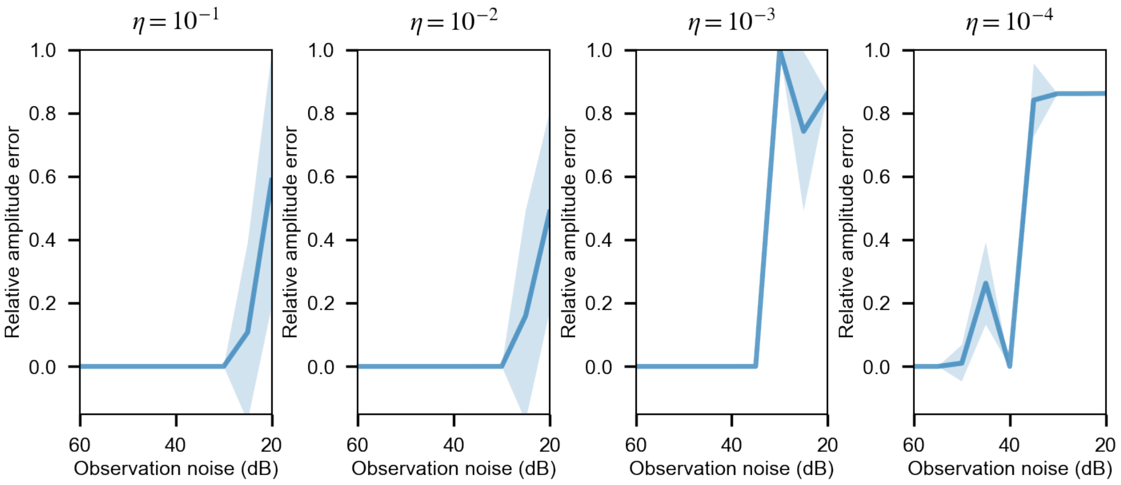

Supplementary Fig. S6: Relative error of the steady state oscillation amplitude under additive Gaussian white noise for different values of the weak nonlinear magnitude η

Supplementary Fig. S6 shows the relative error of the steady state amplitude under different noise levels and for different values of the weak nonlinearity parameter η. The results show that when the correct model structure is recovered, the amplitude error remains small. When the identification fails, the amplitude error becomes large. This demonstrates that the steady state amplitude provides a meaningful indicator of dynamical fidelity under noisy observations. The error is strongly correlated with whether the correct model structure is recovered. Only when the structural identification is successful does the learned system reproduce the correct dynamical behavior.

3.3 Quantification of inference inaccuracy

To quantify the difference between the true equation and inferred equation, we introduce symmetric mean absolute percentage error (sMAPE)

$$\text{sMAPE} = \frac{1}{k}\sum_{i=0}^{k}\frac{|I_i - R_i|}{|I_i + R_i|} \tag{S16}$$

where k is the cardinal number of the set containing the inferred and true terms, I_i and R_i are the inferred and true coefficients for each term respectively. The value of sMAPE lies within the interval from 0 to 1, where smaller values indicate more accurate inference results. Importantly, sMAPE is sensitive to both false negative and false positive errors. For example, if the inferred equation contains a term that should not be present, or misses a term that should be included, the sMAPE value will increase significantly.

4 Details of Comparison experiments

4.1 Description of baseline methods

Here we briefly describe the related methods on inferring weakly nonlinear systems for comparison purpose.

SINDy-STLSQ[1]: the sparse identification of nonlinear dynamics(SINDy) with sequentially thresholded least squares regression. In the comparative testing, we use implementations provided by PySINDy[2].

SINDy-SR3[3]: SINDy with sparse relaxed regularized regression. In the comparative testing, we use implementations provided by PySINDy[2].

ENS-SINDy[4]: Ensemble-SINDy leverage the statistical approach of bootstrap aggregating (bagging) to robustify equation identification mission. In the comparative testing, we use implementations provided by PySINDy[2].

Symbolic regression(SR)[?] : a type of machine learning which aims to discover human-interpretable symbolic models. SR is different from traditional regression that aims at finding a linear or polynomial combination of the input variables with optimal coefficients. The core idea of SR is that any equation can be expressed by a binary tree, in which the leaves are variables and each branching point is an operator. In the comparative testing, we use implementations provided by PySR[5].

UQ-SINDy[6]: uncertainty quantification SINDy, a probabilistic model discovery method for identifying ordinary differential equations governing the dynamics of observed multivariate data. UQ-SINDy promotes robustness against observation noise and limited data, interpretability (in terms of model selection and inclusion probabilities) and generalization capacity for out-of-sample forecast. In the comparative testing, we use implementations provided by `https://github.com/sethhirsh/BayesianSindy`.

Modified-SINDy[7]: a variant of the SINDy algorithm that integrates automatic differentiation and recent time-stepping constrained for simultaneously (1) denoising the data, (2) learning and parametrizing the noise probability distribution, and (3) identifying the underlying parsimonious dynamical system responsible for generating the time-series data. In the comparative testing, we use implementations provided by `https://github.com/dynamicslab/modified-SINDy`.

DySMHO[8]: a machine learning framework rooted in moving horizon nonlinear optimization for identifying governing equations in the form of ordinary differential equations from noisy experimental data sets. In the comparative testing, we use implementations provided by `https://github.com/Baldea-Group/DySMHO`.

DAHSI[9]: data assimilation for hidden, sparse inference, a method combining variational annealing with sparse-optimization methods to perform model identification for chaotic systems with unmeasured variables. In the comparative testing, we use implementations provided by `https://github.com/hribera/DAHSI`.

SIDDs[10]: an algorithm for the simultaneous identification and denoising of a dynamical system (SIDDS). SIDDs infers the noise in the state measurements by requiring that the denoised state satisfies the dynamical system with an equality constraint. In the comparative testing, we use implementations provided by `https://https://github.com/jeffrey-hokanson/SIDDS`.

4.2 Details of weakly nonlinear benchmark

In this Section, we build a weakly nonlinear benchmark test considering observational noise and magnitude of the weak nonlinearities. To explain the advantages of EvLOWN as opposed to other dynamics inference

Supplementary Tab. S4: Simulation parameters in the comparative experiment

η	Time span
1.00×10^{-1}	100
4.64×10^{-2}	300
2.15×10^{-2}	600
1.00×10^{-2}	1000
4.64×10^{-3}	2000
2.15×10^{-3}	5000
1.00×10^{-3}	10000
4.64×10^{-4}	25000
2.15×10^{-4}	50000
1.00×10^{-4}	100000

methods, we develop a comparative experiment on a Van der Pol oscillator:

$$\begin{aligned} \ddot{x} + 10x + \eta(2x^2 - 1)\dot{x} = 0 \\ x^{obs} = x + aN(t) \end{aligned} \tag{S17}$$

where η is a scale factor of weakly non-linear terms and a is a multiplicative parameter of a white Gaussian noise, with $N(t)$ normal distribution with zero mean and standard deviation. Eq.(S17) has been simulated using a fourth-order Runge-Kutta model with fixed time step size. In the paper we generate observational noisy using Python and characterize the noisy data with specified Signal-to-Noise Ratio(SNR). To simulate observational noise, we add Gaussian noise to the displacement data and quantify the noise intensity using the signal-to-noise ratio. To analyze the effects of different magnitudes of weak nonlinear terms, we multiply them by a scaling coefficient η and extend the simulation time to capture the entire evolution process (Supplementary Table 4). For each experiment, we generate 100 independent datasets and recover the underlying governing equations with each algorithm.

The comparative experiments span over 9 different intensities $a \in [1 \times 10^{-4}, 1 \times 10^{-1}]$ (Log-Linear Sampling) and 10 different coefficients $\eta \in [60dB, 20dB]$ (Linear Sampling).

In order to capture the entire evolution process, the simulated time span varies according to η, as listed in the Supplementary Table S4. The hyperparameters of EvLOWN are selected as follows: the residual tolerance for orthogonal matching pursuit is set to 1×10^{-3}, while the increment of residual tolerance for orthogonal matching pursuit is 1×10^{-4}, the weight ratio is 5 and the lowest contribution ratio is 0.5.

For a fair comparison of algorithmic performance, we assume that SINDy has prior knowledge of the approximate magnitude of the weak nonlinear term, with its cut-off parameter consistently set to 0.5η in order to guarantee that weak non-linear terms in the inferred equation are not omitted a-priori.

4.3 Inference results

In the following we describe in detail the simulations of data with observational noise, and show the failure ratio of inferring the structure of true elementary functions out of 100 independent runs in Supplementary Fig. S7.

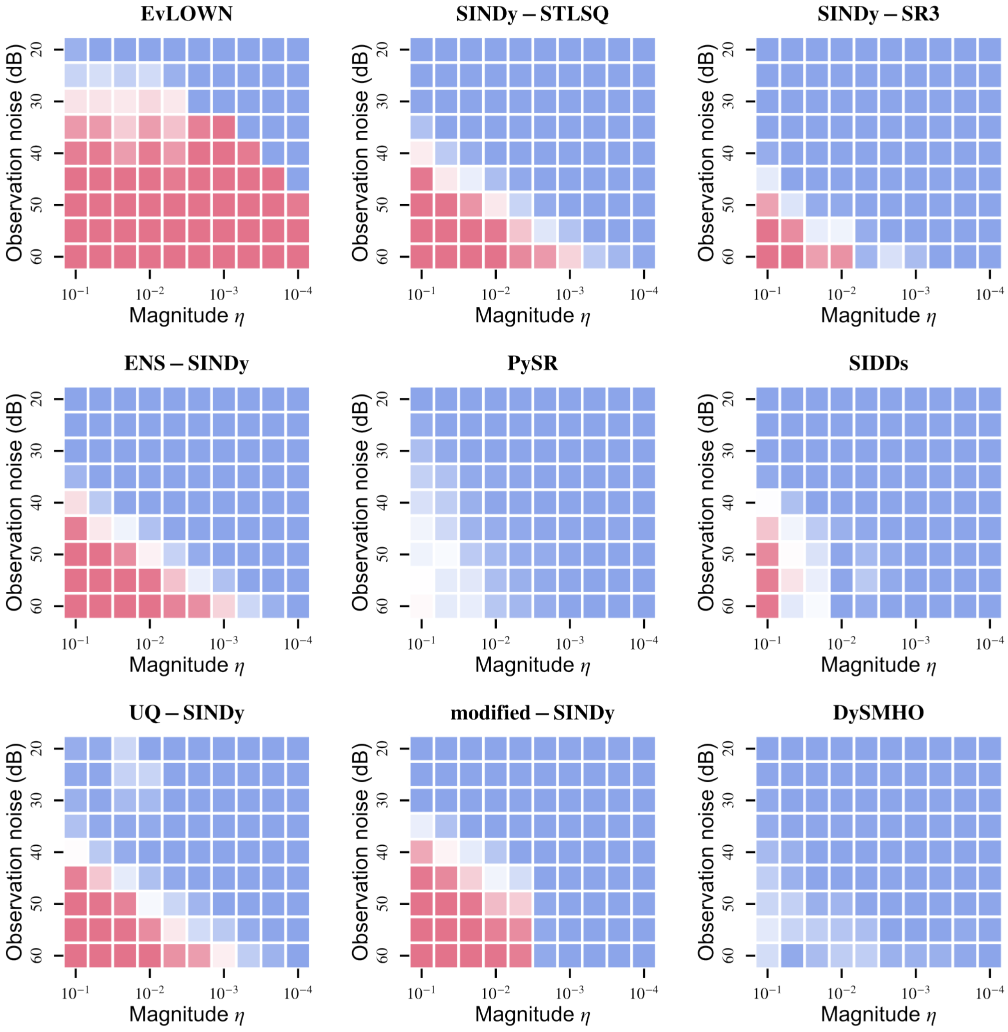

Supplementary Fig. S7: Comparative evaluation of robustness against observational noise and weak nonlinearity strength across several state-of-the-art methods. Color in the heatmaps indicates the success rate of correctly identifying the structure of the true governing equations over 100 independent runs.

4.4 Discussion about DAHSI

DAHSI is designed for model discovery in systems that exhibit chaotic behavior and contain hidden or unmeasured variables. The method combines variational annealing, originally developed for parameter estimation in chaotic dynamical systems, with sparse optimization techniques for model selection. Time-delay embeddings are first used to reconstruct a manifold representation of the underlying attractor when only a subset of the state variables is observable. Within this reconstructed state space, DAHSI employs sparse optimization over a candidate function library to identify a parsimonious set of governing equations that is consistent with the observed dynamics.

Although DAHSI is powerful for chaotic systems with partially observed states, several limitations arise when it is applied to weakly nonlinear oscillatory systems. First, the variational annealing procedure involves solving a high-dimensional nonlinear optimization problem that is sensitive to initial guesses and algorithmic hyperparameters. This results in substantial computational cost and nontrivial tuning requirements. Second, the identification accuracy of DAHSI relies on the quality of time-delay embeddings. For weakly nonlinear systems, the dynamical signatures accumulate slowly and are subtle in the

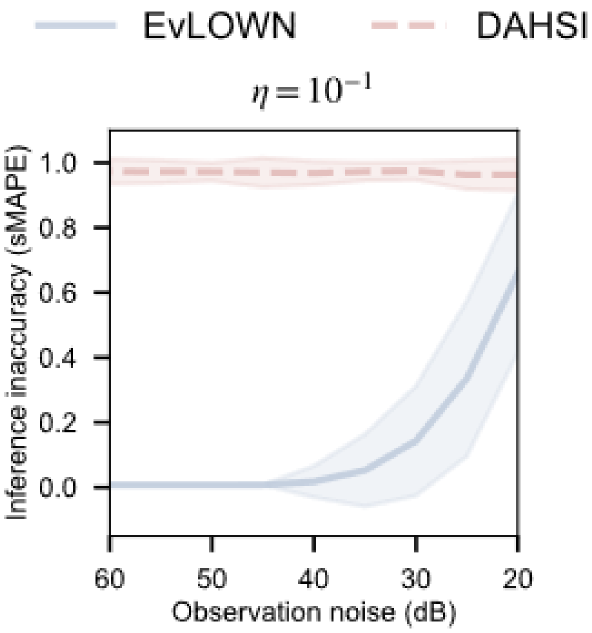

Supplementary Fig. S8: Inference inaccuracy of DAHSI compared to EvLOWN at $\eta = 0.1$ magnitude in different observational noise

reconstructed manifold, which makes it difficult to distinguish weak nonlinear terms from embedding and reconstruction errors. Third, DAHSI ultimately performs sparse regression in the reconstructed state space, and the sparsity-promoting thresholding tends to eliminate weak but dynamically essential terms when their contributions are comparable to embedding errors and residual noise. As a consequence, although DAHSI is suitable for chaotic systems with strong nonlinear interactions, its reliability and efficiency are limited in the weakly nonlinear regime considered in our benchmark study.

DAHSI is not included in the full benchmark sweep because, in practical implementations, the optimization problem treats not only the model parameters but also the entire trajectory as decision variables. Each trajectory point is optimized as part of the moving-horizon formulation. As a result, the computational cost grows very rapidly with the number of samples, and the method becomes increasingly slow as the sequence length increases.

For this reason, we restricted our evaluation to the simplest weak-nonlinearity magnitude of 10^{-1} and considered nine different noise levels. Within this reduced setting, DAHSI was tested on 9×100 independent trials. In all of these trials, the method did not succeed in identifying the correct governing equation. The figure reports the corresponding coefficient identification errors across different noise levels. The results confirm that, although DAHSI is conceptually capable of handling systems with hidden variables and chaotic dynamics, the combination of high computational complexity and the large number of optimization degrees of freedom severely limits its practical performance in weakly nonlinear systems.

5 Satellite orbit dynamics

The space station orbit data used in this study are sourced from the publicly available Orbital Ephemeris Message (OEM). OEM is one of the three standard file formats defined by the Consultative Committee

Supplementary Tab. S5: The testing trajectories information of CSS

Trajectory	Start time	End time
Jan	2024/1/8 00:00	2024/1/15 00:00
Feb	2024/2/5 00:00	2024/2/12 00:00
Mar	2024/3/4 00:00	2024/3/11 00:00
Apr	2024/4/1 00:00	2024/4/8 00:00
May	2024/5/6 00:00	2024/5/13 00:00
Jun	2024/6/3 00:00	2024/6/10 00:00
Jul	2024/7/1 00:00	2024/7/8 00:00
Sep	2024/9/2 0:00	2024/9/9 0:00
Oct	2024/10/7 0:00	2024/10/14 0:00
Nov	2023/11/15 0:00	2023/11/22 0:00
Dec	2023/12/4 0:00	2023/12/11 0:00

for Space Data Systems (CCSDS) for transmitting spacecraft orbit information. These files provide the position and velocity of a given object at multiple epochs. Currently, there are two fully operational space stations: the International Space Station (ISS) and China's Tiangong Space Station (CSS). We apply EvLOWN to infer the governing equations from the OEM data of the ISS[11] and CSS[12]. The inferred equations for the ISS and CSS share the same structure, differing only slightly in their coefficients. More details are presented in this section.

The hyperparameters of EvLOWN are selected as follows: the residual tolerance for orthogonal matching pursuit is 1×10^{-1}, the increment of residual tolerance is 1×10^{-1}, the lowest contribution ratio is 0.1 and the weight ratio is 10. A group of 33 candidate basis functions $\Theta \in \mathbb{R}^{1\times 33}$ is used to reconstruct the ODE, consisting of polynomial terms up to third order .

5.1 China's Tiangong Space Station

The training dataset used for inferring CSS governing equations consists of orbital ephemeris messages from August 5th, 2024, to August 12th, 2024. This dataset captures the three-dimensional motion and velocity of the CSS in the J2000 reference frame, recorded at 4-minute intervals. Applying our approach to the satellite trajectory data, The equation inferred by EvLOWN are shown as:

$$\begin{aligned} \ddot{x}_1 + 16.7617x_1 + 8.9080 \times 10^{-3}\dot{x}_2 &= 0 \\ \ddot{x}_2 + 16.7617x_2 - 8.9690 \times 10^{-3}\dot{x}_1 &= 0 \\ \ddot{x}_3 + 16.7617x_3 &= 0 \end{aligned} \tag{S18}$$

To validate the inferred equations, we selected 11 trajectories from time periods different from those of the training set, ranging from January to December (Supplementary Table S5). We integrated the inference equation (Eq.S18) from the initial conditions of each test trajectory to obtain 11 inferred trajectories. The comparison between true trajectories and inferred trajectories are shown in Main text. This verifies that the EvLOWN method learn the orbital dynamics of the space station orbit. The uncertainty measurement of inferred coefficients are listed in Supplementary Table S6.

Supplementary Tab. S6: Statistical analyzes of uncertainty in the inferred coefficients of CSS orbit dynamics

	Relevant Term	Mean Value	Standard Deviation	95% Confidence intervals
$\ddot{x}_1 = f_1(x_1, x_2, x_3)$	x_1	-16.3871	$2.1316 \cdot 10^{-12}$	$(-16.3871, -16.3871)$
	$\dot{x}_2$	$-8.8799 \cdot 10^{-3}$	$1.1956 \cdot 10^{-5}$	$(-8.9033 \cdot 10^{-3},\ -8.8564 \cdot 10^{-3})$
$\ddot{x}_2 = f_2(x_1, x_2, x_3)$	x_2	-16.3871	$2.1316 \cdot 10^{-12}$	$(-16.3871, -16.3871)$
	$\dot{x}_1$	$8.9507 \cdot 10^{-3}$	$5.2071 \cdot 10^{-5}$	$(8.8486 \cdot 10^{-3}, 9.0527 \cdot 10^{-3})$
$\ddot{x}_3 = f_3(x_1, x_2, x_3)$	x_3	-16.3871	$2.1316 \cdot 10^{-12}$	$(-16.3871, -16.3871)$

Supplementary Tab. S7: The testing trajectories information of ISS

Trajectory	Start time	End time
Jan	2024/1/1 12:00	2024/1/8 12:00
Feb	2024/2/3 12:00	2024/2/10 12:00
Mar	2024/3/4 12:00	2024/3/11 12:02
Apr	2024/4/3 12:00	2024/4/10 12:02
May	2024/5/1 12:00	2024/5/7 12:02
Jun	2024/5/31 12:00	2024/6/6 12:02
Jul	2024/7/1 12:00	2024/7/7 12:00
Sep	2024/9/6 12:02	2024/9/13 12:00
Oct	2024/10/5 12:02	2024/10/12 12:00
Nov	2023/11/1 12:00	2023/11/7 12:00
Dec	2023/12/1 12:00	2023/12/7 12:00

5.2 International Space Station

The training dataset used for inferring ISS governing equations consists of orbital ephemeris collected from August 2nd, 2024, to August 10th, 2024. This dataset also captures the three-dimensional motion and velocity of the ISS in the J2000 reference frame, recorded at 4-minute intervals. Applying our approach to the satellite trajectory data, EvLOWN infers the equations shown in Eq.(S19). It has the same equation form, but a little difference on coefficients with Eq.(S18). The testing dataset are 12 different trajectories of ISS in different time periods (Supplementary Table S7). The trajectories of inferred equations are compared with OEM ground truth in Main text. The uncertainty measurement of inferred coefficients are listed in Supplementary Table S8.

$$\begin{aligned} \ddot{x}_1 + 16.3944x_1 + 7.1969 \times 10^{-3}\dot{x}_2 = 0 \\ \ddot{x}_2 + 16.3944x_2 - 7.1995 \times 10^{-3}\dot{x}_1 = 0 \\ \ddot{x}_3 + 16.3944x_3 = 0 \end{aligned} \tag{S19}$$

6 Vortex-induced vibration case

The wind tunnel test of the physical model of the Xihoumen bridge is carried out on the standard section of the main beam. The main purpose of the wind tunnel test is to collect the vibration data of the main beam of Xihoumen Bridge under different wind speeds and observe the possible vortex-induced vibration

Supplementary Tab. S8: Statistical analyzes of uncertainty in the inferred coefficients of ISS orbit dynamics

	Relevant Term	Mean Value	Standard Deviation	95% Confidence intervals
$\ddot{x}_1 = f_1(x_1, x_2, x_3)$	x_1	-16.3888	$7.1054 \cdot 10^{-15}$	$(-16.3888, -16.3888)$
	$\dot{x}_2$	$-7.1987 \cdot 10^{-3}$	$1.7618 \cdot 10^{-5}$	$(-7.1641 \cdot 10^{-3},\ -7.2332 \cdot 10^{-3})$
$\ddot{x}_2 = f_2(x_1, x_2, x_3)$	x_2	-16.3888	$7.1054 \cdot 10^{-15}$	$(-16.3888, -16.3888)$
	$\dot{x}_1$	$7.2008 \cdot 10^{-3}$	$1.9354 \cdot 10^{-5}$	$(-7.1629 \cdot 10^{-3}, -7.2387 \cdot 10^{-3})$
$\ddot{x}_3 = f_3(x_1, x_2, x_3)$	x_3	-16.3888	$7.1054 \cdot 10^{-15}$	$(-16.3888, -16.3888)$

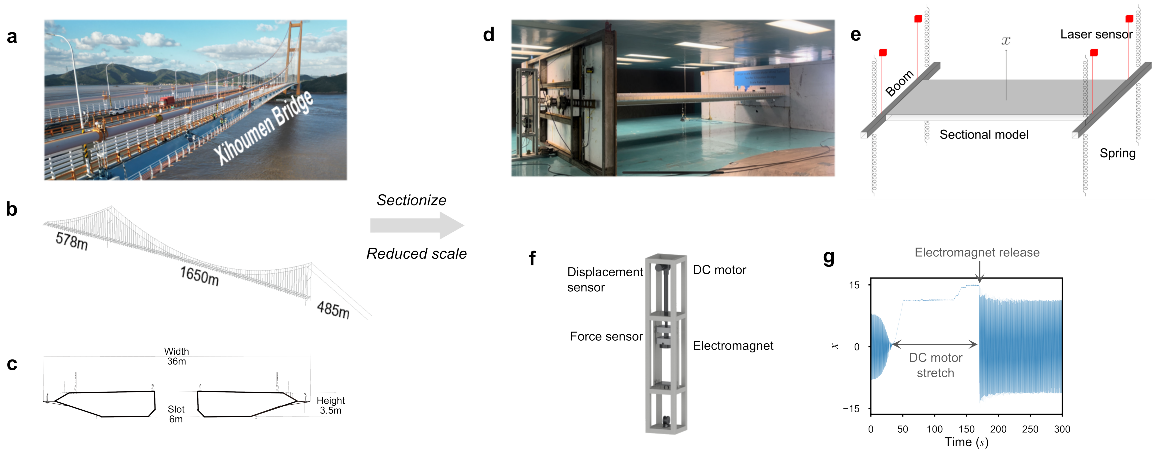

Supplementary Fig. S9: The wind tunnel experiment settings of Xihoumen bridge (a–c) The real suspension bridge in eastern China prone to VIV: (a) Xihoumen Bridge, (b) elevation view, (c) cross-sectional shape of the main beam. (d–h) Wind tunnel experiments on a 1:20 scale model of the Xihoumen bridge deck: (d) TJ-3 boundary wind tunnel facility, (e) photo of the wind tunnel setup, (f) schematic showing the sectional model supported by eight springs simulating bridge stiffness, (g) initial state control device, (h) schematic of the initial state control process.

phenomenon. Xihoumen Bridge is in Zhoushan, Zhejiang Province in mainland China. The bridge is located at the mouth of the Qiantang River on the East Coast of China (30.062°N, 121.906°E). The main bridge is a continuous steel-slotted-box-girder suspension bridge, whose spans are 578m, 1650m, 485m. The main girder comprises a separated steel-box-girder section with a central slot.

6.1 Wind tunnel experiment setup

Scaled model wind tunnel testing is based on the similarity criterion [13]. The wind tunnel test of the scaled sectional model only simulates the vibration characteristics of the two degrees of freedom in the vertical direction and the torsional direction, and the vibration characteristics in the horizontal flow direction (the resistance direction) are not simulated. The wind tunnel test uses a spring-suspended two-dimensional rigid body segment model, which is suspended on a bracket in the tunnel by 8 springs. In addition to matching the geometric similarity, the spring-suspended two-dimensional rigid body segment model wind tunnel test should also match the similarity of the following set of principle shown in Supplementary Table S9.

The wind tunnel experiment settings of Xihoumen bridge are shown in Supplementary Fig. S9. The scaled segment model of the main beam of Xihoumen Bridge is designed according to the standard section of

Supplementary Tab. S9: The similarity principle of scaled sectional bridge model in wind tunnel test

Parameter Name	Symbol	Unit	Scale ratio	similarity principle
Length	L	m	$\lambda_L = 1:20$	Geometry
Wind velocity	U	m/s	$\lambda_U = \lambda_L/\lambda_T$	Strouhal number
Density	ρ	kg/m^3	$\lambda_\rho = 1$	Invariable material
Linear mass	m_m	kg/m	$\lambda_m = \lambda_\rho \lambda_L^2 = 1:20^2$	Dimension analysis
Linear mass moment of inertia	J_m	$kg \cdot m^2/m$	$\lambda_J = \lambda_m \lambda_L^2 = 1:20^4$	Dimension analysis
Time	T	s	$\lambda_T = \lambda_L/\lambda_U$	Strouhal number

Supplementary Tab. S10: Design parameters for the scaled sectional model

Parameter Name	Symbol	Unit	Value of real bridge	Scale ratio	Value of scaled model
Length	L	m	-	$\lambda_L = 1:20$	6.000
Width	B	m	36.0	$\lambda_L = 1:20$	1.800
Height	H	m	3.5	$\lambda_L = 1:20$	0.175
Wind velocity	U	m/s	-	$\lambda_U = 1:1$	-
Linear mass	m_m	kg/m	27854	$\lambda_m = 1:20^2$	69.635
Linear mass moment of inertia	J_m	$kg \cdot m^2/m$	3581200	$\lambda_J = 1:20^4$	22.383
Vertical oscillating frequency	f_v	Hz	0.1002	$1/\lambda_T = 20:1$	2.040
Torsional oscillating frequency	f_t	Hz	0.2335	$1/\lambda_T = 20:1$	4.670

mid-span, and the geometric scale ratio is 1:20. The scaled model is 6.000m long and 1.800m wide, and the ratio of the segment model length to width is 6.000/1.800=3.33; the maximum height of the model box is 0.175m (the maximum blocking rate of the box is 0.175/2=8.75%). In order to make the segment model have a larger Reynolds number and increase the natural frequency of the model, the wind speed ratio is taken as 1:1, that is, the test wind speed is close to the actual bridge wind speed. More parameters of scaled sectional model are shown in Supplementary Table S10.

The wind tunnel test of the scaled sectional model vortex-induced vibration was carried out in the TJ-3 atmospheric boundary layer wind tunnel of Tongji University. The TJ-3 atmospheric boundary layer wind tunnel is a closed re-flow wind tunnel. The test section is 14 m long, with a rectangular cross-section 15 m wide and 2 m high. The TJ-3 wind tunnel body consists of 1 drive section, 2 diffuser sections, 1 stabilizer section, 1 damping net, 1 contraction section, and 1 test section. The wind tunnel power system is driven by seven 45-kilowatt DC motors, and the fan system consists of 8 fiberglass blades. The wind velocity range of the wind tunnel is 1.0 - 17.6 m/s, which is continuously adjustable. The main measurement objects of the wind tunnel test are wind velocity and displacement of model. The reference wind speed of the test flow field is measured by a pitot tube and a micro-manometer, and the height of the measuring point is consistent with the height of the bridge deck. The displacement of model is measured by a laser displacement meter. Four laser displacement meters are arranged on the left and right sections of the segment model, which can measure the vertical and torsional displacements of the

segment model at the same time. The sampling frequency of the vibration signal is 300Hz.

In order to obtain data with different initial states, we develop an initial state control device. It pulls the model through a DC motor and releases the constraints by controlling the electromagnet to make the model start to vibrate. In order to quantitatively control the initial displacement of the model, the device is equipped with a force sensor and a displacement sensor. Here we show the average results of the upper and lower springs in Supplementary Figure S10. The theoretical system vertical stiffness of 8 the supporting springs is $4 \times (15.8625 + 2.5925) = 73.82N/mm$. Further, the theoretical vertical oscillating circular frequency is $\omega_{tv} = \sqrt{73820/(6 \times 69.635)} = 176.68$, which is close to our inferred equations using different wind speeds. The errors might be associated to additional masses of the model, resulting in the actual mass of the model being larger than the designed value.

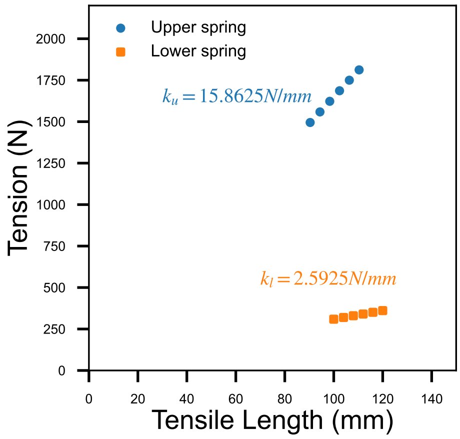

Supplementary Fig. S10: Tension test of supporting springs

6.2 Inferring results for other wind speeds

In Figure 5c of the main text, we show the stable oscillation amplitudes versus wind velocities; 8 different wind speed experiments were recorded, 5 of which showed vortex-induced vibration and 3 were just free vibrations. The hyper-parameters of EvLOWN are listed in Supplementary Table S11.

This section shows the identification results of the dynamic equations for wind speeds other than those in Figure 5d, e in the main text. The inferred results for free vibrations at wind speed $U = 2.16m/s, 3.00m/s$ are shown in Supplementary Figure S11. The inferred results for vortex-induced vibration at wind speed $U = 2.48m/s, 2.60m/s, 2.73m/s, 2.82m/s$ are shown in Supplementary Figure S12.

6.3 Influence of weak terms

In Figure 5 of the main text we present the results of the inferred equations for free vibration ($U = 0.00m/s$) and vortex-induced vibration ($U = 2.54m/s$) experimental data. Interestingly, there are some terms with very small coefficients which are usually considered to be negligible in standard approaches. To illustrate the contribution of these terms to the dynamical system, we perform two sets of ablation experiments in which we observe the fitting results obtained by removing the terms with small coefficients respectively to determine their impact on the dynamic system. Supplementary Figure S13 and S14 show the results of ablation experiments about free vibration case and vortex-induced vibration case

Supplementary Tab. S11: The hyper-parameter selection of EvLOWN in vortex-induced vibration case

Wind Velocity	Phenomenon	residual tolerance	increment of residual tolerance	lowest contribution ratio	Weight ratio
0.00m/s	Free Vibration	1×10^{-3}	1×10^{-4}	0.01	1
2.16m/s	Free Vibration	1×10^{-3}	1×10^{-4}	0.01	1
2.48m/s	Vortex-induced Vibration	1×10^{-3}	1×10^{-3}	0.015	1
2.54m/s	Vortex-induced Vibration	1×10^{-3}	1×10^{-3}	0.015	1
2.60m/s	Vortex-induced Vibration	1×10^{-3}	1×10^{-3}	0.015	1
2.73m/s	Vortex-induced Vibration	1×10^{-3}	1×10^{-3}	0.015	1
2.82m/s	Vortex-induced Vibration	1×10^{-3}	1×10^{-3}	0.015	1
3.00m/s	Free Vibration	1×10^{-3}	1×10^{-4}	0.01	1

respectively. The colors represent the terms with small coefficient in the inferred equation.

Firstly, we quantitatively compute the relative error (defined in Eq.(S20)) under different ablated terms. It can be seen that removing the term with small coefficients will not lead to obvious errors in the fitting of differential equations $\ddot{x}$, and the relative errors (defined as Eq. (S20)) are all less than 0.02 (Supplementary Figure S13a and Supplementary Figure S14a). However, when this differential equation is used to integrate from the same initial states, it deviates significantly from the observations (Supplementary Figure S13b and Supplementary Figure S14b). To further explain how these terms with small coefficients affects dynamics, we compute the evolutionary variables of different ablated terms and make a comparison in Supplementary Figure S13c,d and Supplementary Figure S14c,d. The differences between inferred equation with ablated terms and observations in time domain are shown in Supplementary Figure S13e and Supplementary Figure S14e. The ablation studies prove that although removing any small coefficient term has almost no effect on the fitting of the differential equation, it has a great impact on the long-term evolution of the dynamic system. EvLOWN can find all significant small coefficient terms to rebuild the true dynamics.

$$R = \frac{||\hat{\boldsymbol{y}} - \boldsymbol{y}||_2}{||\boldsymbol{y}||_2} \tag{S20}$$

where $||\cdot||_2$ is the normalization operator L_2, $\boldsymbol{y}$ is the true value of dynamics or derivatives, and $\hat{\boldsymbol{y}}$ is the prediction value.

References

[1] Steven L Brunton, Joshua L Proctor, and J Nathan Kutz. Discovering governing equations from data by sparse identification of nonlinear dynamical systems. *Proc. Natl. Acad. Sci.*, 113(15):3932–3937, 2016.

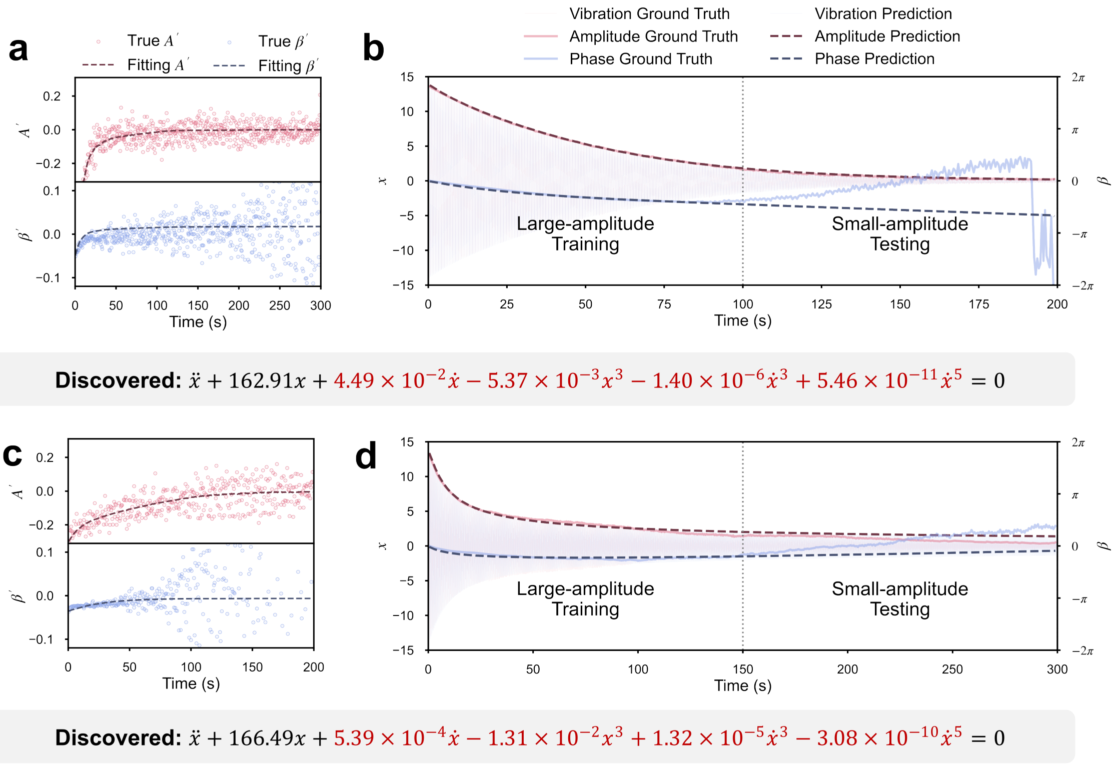

Supplementary Fig. S11: Inferred results of free vibration at wind velocity $U = 2.16m/s$(a-b) and $U = 3.00m/s$ (c-d): a,c. Evolutionary ODE discovery and fitting ; b,d. Comparison between inferred equation and observations on dynamics and evolutionary variables

Supplementary Tab. S12: Statistical analyzes of uncertainty in the inferred coefficients of bridge section free vibration in wind tunnel

Wind velocity	Relevant Term	Mean Value	Standard Deviation	95% Confidence intervals
$0.00m/s$	x	$-1.6292 \cdot 10^2$	$2.1111 \cdot 10^{-1}$	$(-1.6333 \cdot 10^2, -1.6251 \cdot 10^2)$
	x^3	$1.0810 \cdot 10^{-2}$	$4.3463 \cdot 10^{-3}$	$(2.2907 \cdot 10^{-3}, 1.9328 \cdot 10^{-2})$
	$\dot{x}$	$-3.1018 \cdot 10^{-2}$	$2.5273 \cdot 10^{-3}$	$(-3.5971 \cdot 10^{-2}, -2.6064 \cdot 10^{-2})$
	$\dot{x}^3$	$-3.7406 \cdot 10^{-6}$	$6.8565 \cdot 10^{-7}$	$(-5.0844 \cdot 10^{-6}, -2.3967 \cdot 10^{-6})$
	$\dot{x}^5$	$9.0233 \cdot 10^{-11}$	$3.2011 \cdot 10^{-11}$	$(2.7493 \cdot 10^{-11}, 1.5297 \cdot 10^{-10})$
$2.16m/s$	x	$-1.6294 \cdot 10^2$	$9.2889 \cdot 10^{-2}$	$(-1.6312 \cdot 10^2, -1.6275 \cdot 10^2)$
	x^3	$5.4720 \cdot 10^{-3}$	$2.4486 \cdot 10^{-3}$	$(6.7277 \cdot 10^{-4}, 1.0271 \cdot 10^{-2})$
	$\dot{x}$	$-4.4916 \cdot 10^{-2}$	$2.6806 \cdot 10^{-3}$	$(-5.0170 \cdot 10^{-2}, -3.9662 \cdot 10^{-2})$
	$\dot{x}^3$	$1.3788 \cdot 10^{-6}$	$4.8650 \cdot 10^{-7}$	$(4.2530 \cdot 10^{-7}, 2.3323 \cdot 10^{-6})$
	$\dot{x}^5$	$-5.3160 \cdot 10^{-11}$	$1.8094 \cdot 10^{-11}$	$(-8.8625 \cdot 10^{-11}, -1.7696 \cdot 10^{-11})$
$3.00m/s$	x	$-1.6644 \cdot 10^2$	$1.4693 \cdot 10^{-1}$	$(-1.6673 \cdot 10^2, -1.6615 \cdot 10^2)$
	x^3	$1.2017 \cdot 10^{-2}$	$4.0456 \cdot 10^{-3}$	$(4.0880 \cdot 10^{-3}, 1.9947 \cdot 10^{-2})$
	$\dot{x}$	$-8.6965 \cdot 10^{-4}$	$2.5684 \cdot 10^{-3}$	$(-5.9036 \cdot 10^{-3}, 4.1643 \cdot 10^{-3})$
	$\dot{x}^3$	$-1.2982 \cdot 10^{-5}$	$1.0046 \cdot 10^{-6}$	$(-1.4951 \cdot 10^{-5}, -1.1013 \cdot 10^{-5})$
	$\dot{x}^5$	$2.9907 \cdot 10^{-10}$	$5.6080 \cdot 10^{-11}$	$(1.8916 \cdot 10^{-10}, 4.0899 \cdot 10^{-10})$

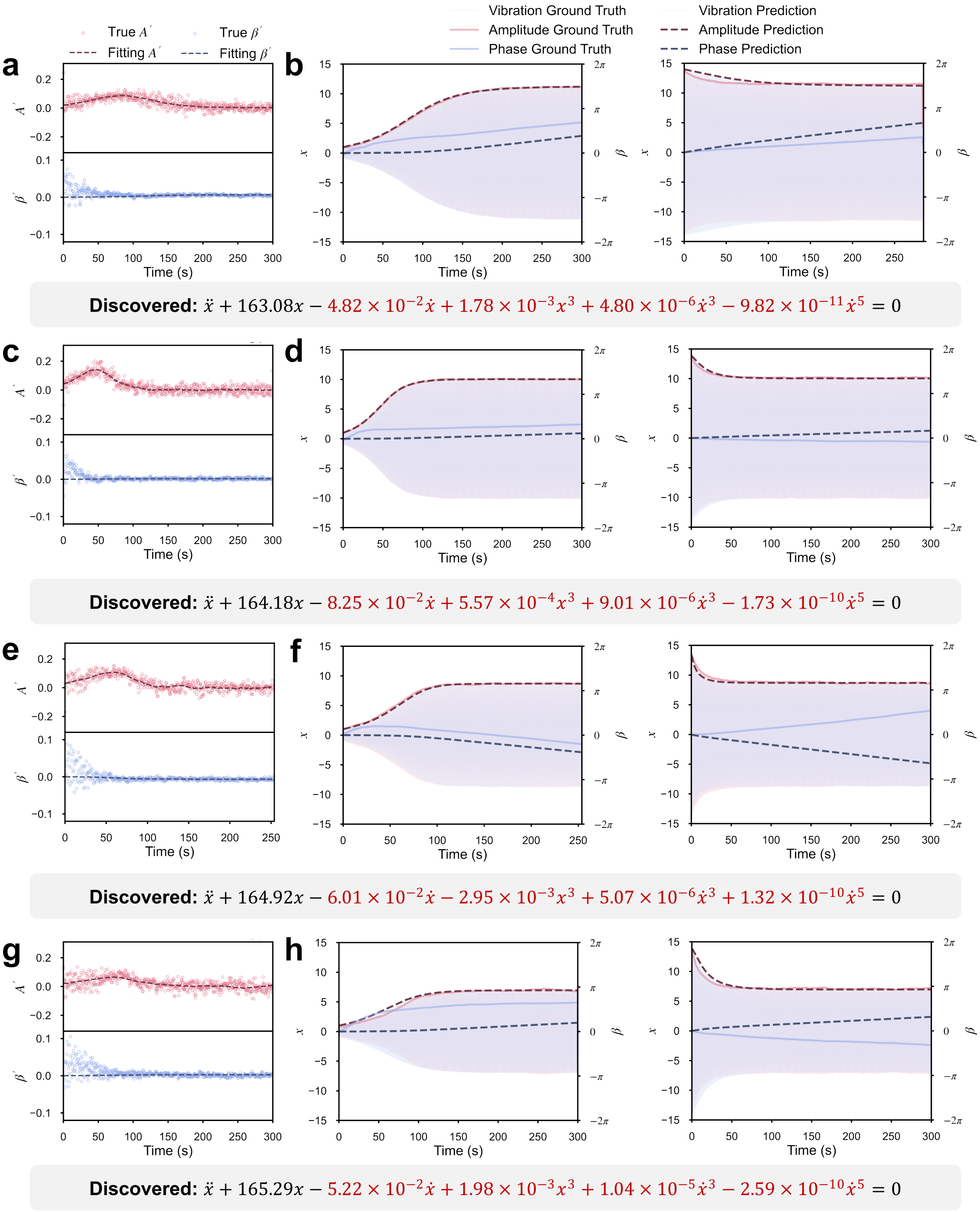

Supplementary Fig. S12: Inferred results of vortex-induced vibration at wind velocity $U = 2.48m/s$(a-b), $U = 2.60m/s$(c-d), $U = 2.72m/s$(e-f) and $U = 2.81m/s$ (g-h): a,c,e,g. Evolutionary ODE discovery and fitting ; b,d,f,h. Comparison between inferred equation and observations on dynamics and evolutionary variables

Supplementary Tab. S13: Statistical analyzes of uncertainty in the inferred coefficients of bridge section vortex-induced vibration in wind tunnel

Wind velocity	Relevant Term	Mean Value	Standard Deviation	95% Confidence intervals
$2.48m/s$	x	$-1.6308 \cdot 10^{2}$	$3.1264 \cdot 10^{-13}$	$(-1.6308 \cdot 10^{2}, -1.6308 \cdot 10^{2})$
	x^3	$-3.8300 \cdot 10^{-3}$	$2.3212 \cdot 10^{-3}$	$(-8.3794 \cdot 10^{-3}, 7.1951 \cdot 10^{-4})$
	$\dot{x}$	$4.8104 \cdot 10^{-2}$	$1.1815 \cdot 10^{-3}$	$(4.5788 \cdot 10^{-2}, 5.0420 \cdot 10^{-2})$
	$\dot{x}^3$	$-4.7865 \cdot 10^{-6}$	$2.8007 \cdot 10^{-7}$	$(-5.3354 \cdot 10^{-6}, -4.2376 \cdot 10^{-6})$
	$\dot{x}^5$	$9.7736 \cdot 10^{-11}$	$1.2760 \cdot 10^{-11}$	$(7.2726 \cdot 10^{-11}, 1.2275 \cdot 10^{-10})$
$2.54m/s$	x	$-1.6308 \cdot 10^{2}$	$3.1264 \cdot 10^{-13}$	$(-1.6308 \cdot 10^{2}, -1.6308 \cdot 10^{2})$
	x^3	$-3.7074 \cdot 10^{-3}$	$2.2274 \cdot 10^{-3}$	$(-8.0731 \cdot 10^{-3}, 6.5831 \cdot 10^{-4})$
	$\dot{x}$	$4.7330 \cdot 10^{-2}$	$1.0569 \cdot 10^{-3}$	$(4.5258 \cdot 10^{-2}, 4.9401 \cdot 10^{-2})$
	$\dot{x}^3$	$-4.6249 \cdot 10^{-6}$	$2.7742 \cdot 10^{-7}$	$(-5.1687 \cdot 10^{-7}, -4.0812 \cdot 10^{-6})$
	$\dot{x}^5$	$9.0739 \cdot 10^{-11}$	$1.2866 \cdot 10^{-11}$	$(6.5522 \cdot 10^{-11}, 1.1596 \cdot 10^{-10})$
$2.60m/s$	x	$-1.6418 \cdot 10^{2}$	$5.6843 \cdot 10^{-14}$	$(-1.6418 \cdot 10^{2}, -1.6418 \cdot 10^{2})$
	x^3	$-2.4690 \cdot 10^{-3}$	$2.1858 \cdot 10^{-3}$	$(-6.7532 \cdot 10^{-3}, 1.8151 \cdot 10^{-2})$
	$\dot{x}$	$8.2514 \cdot 10^{-2}$	$1.8405 \cdot 10^{-3}$	$(7.8907 \cdot 10^{-3}, 8.6122 \cdot 10^{-3})$
	$\dot{x}^3$	$-9.0183 \cdot 10^{-6}$	$4.7427 \cdot 10^{-7}$	$(-9.9479 \cdot 10^{-6}, -8.0888 \cdot 10^{-6})$
	$\dot{x}^5$	$1.7328 \cdot 10^{-10}$	$2.5221 \cdot 10^{-11}$	$(1.2385 \cdot 10^{-10}, 2.2271 \cdot 10^{-10})$
$2.73m/s$	x	$-1.6492 \cdot 10^{2}$	$2.5580 \cdot 10^{-13}$	$(-1.6492 \cdot 10^{2}, -1.6492 \cdot 10^{2})$
	x^3	$2.0938 \cdot 10^{-3}$	$1.1431 \cdot 10^{-3}$	$(-1.4668 \cdot 10^{-4}, 4.3344 \cdot 10^{-3})$
	$\dot{x}$	$6.0383 \cdot 10^{-2}$	$2.6477 \cdot 10^{-3}$	$(5.5193 \cdot 10^{-2}, 6.5572 \cdot 10^{-2})$
	$\dot{x}^3$	$-5.1834 \cdot 10^{-6}$	$9.3645 \cdot 10^{-7}$	$(-7.0189 \cdot 10^{-6}, -3.3480 \cdot 10^{-6})$
	$\dot{x}^5$	$-1.2385 \cdot 10^{-10}$	$6.6220 \cdot 10^{-11}$	$(-2.5364 \cdot 10^{-10}, 5.9438 \cdot 10^{-12})$
$2.82m/s$	x	$-1.6529 \cdot 10^{2}$	$2.8422 \cdot 10^{-13}$	$(-1.6529 \cdot 10^{2}, -1.6529 \cdot 10^{2})$
	x^3	$-1.4433 \cdot 10^{-3}$	$1.4082 \cdot 10^{-2}$	$(-4.2033 \cdot 10^{-2}, 1.3168 \cdot 10^{-2})$
	$\dot{x}$	$5.2219 \cdot 10^{-2}$	$2.4354 \cdot 10^{-3}$	$(4.7445 \cdot 10^{-2}, 5.6992 \cdot 10^{-2})$
	$\dot{x}^3$	$-1.0387 \cdot 10^{-5}$	$1.2120 \cdot 10^{-6}$	$(-7.0189 \cdot 10^{-6}, -8.0119 \cdot 10^{-6})$
	$\dot{x}^5$	$2.5601 \cdot 10^{-10}$	$1.3073 \cdot 10^{-10}$	$(-2.2324 \cdot 10^{-13}, 5.1224 \cdot 10^{-10})$

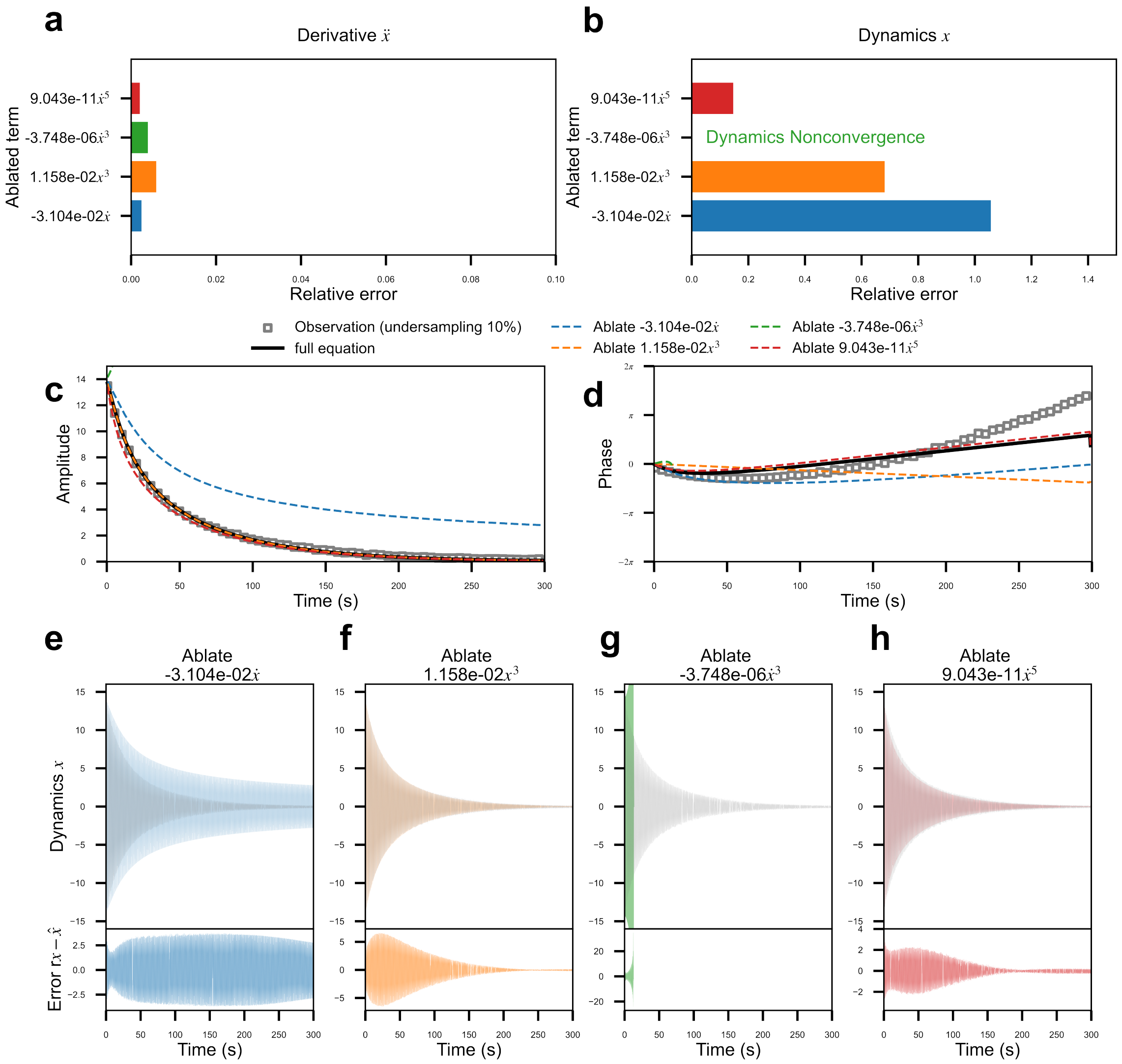

Supplementary Fig. S13: Ablation study for free vibration (Wind velocity $U = 0.00m/s$): (a) Relative error on derivative $\ddot{x}$ fitting under different weak term ablated; (b) Relative error on dynamic x fitting under different weak term ablated; (c) Amplitude matching comparison between different weak term ablated, full inferred equation and observations; (d) Phase matching comparison between different weak terms ablated, full inferred equation and observations; (e-g) Dynamics fitting comparison and error in time domain under different weak term ablated

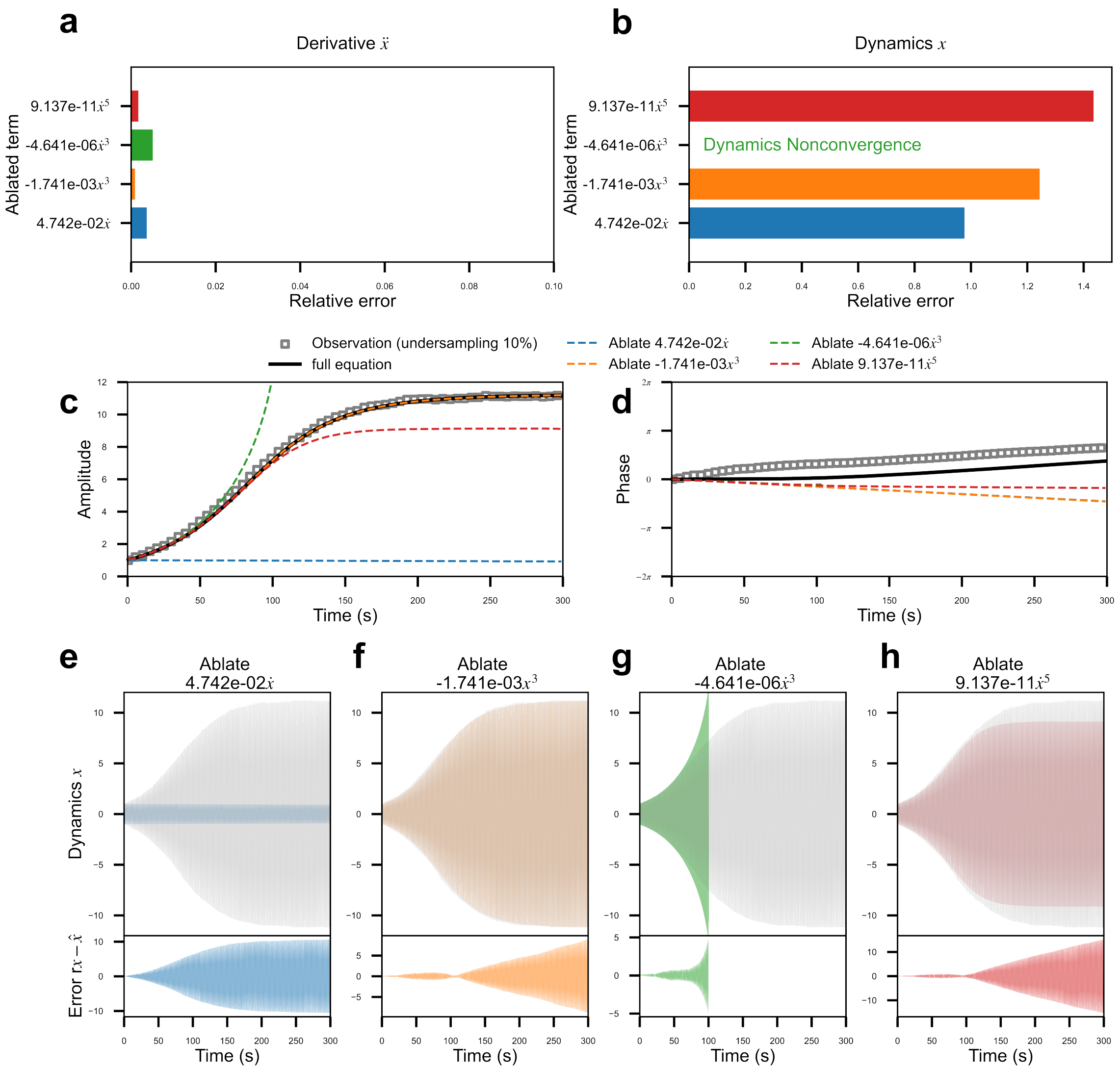

Supplementary Fig. S14: Ablation study for vortex-induced vibration (Wind velocity $U = 2.54m/s$): (a) Relative error on derivative $\ddot{x}$ fitting under different weak term ablated; (b) Relative error on dynamic x fitting under different weak term ablated; (c) Amplitude matching comparison between different weak term ablated, full inferred equation and observations; (d) Phase matching comparison between different weak terms ablated, full inferred equation and observations; (e-h) Dynamics fitting comparison and error in time domain under different weak term ablated

[2] Brian M de Silva, Kathleen Champion, Markus Quade, Jean-Christophe Loiseau, J Nathan Kutz, and Steven L Brunton. Pysindy: a python package for the sparse identification of nonlinear dynamics from data. *arXiv preprint arXiv:2004.08424*, 2020.

[3] Peng Zheng, Travis Askham, Steven L Brunton, J Nathan Kutz, and Aleksandr Y Aravkin. A unified framework for sparse relaxed regularized regression: Sr3. *IEEE Access*, 7:1404–1423, 2018.

[4] Urban Fasel, J Nathan Kutz, Bingni W Brunton, and Steven L Brunton. Ensemble-sindy: Robust sparse model discovery in the low-data, high-noise limit, with active learning and control. *Proc. R. Soc. A*, 478(2260):20210904, 2022.

[5] Miles Cranmer. Interpretable machine learning for science with pysr and symbolicregression. jl. *arXiv preprint arXiv:2305.01582*, 2023.

[6] Seth M Hirsh, David A Barajas-Solano, and J Nathan Kutz. Sparsifying priors for bayesian uncertainty quantification in model discovery. *Royal Society open science*, 9(2):211823, 2022.

[7] Kadierdan Kaheman, Steven L Brunton, and J Nathan Kutz. Automatic differentiation to simultaneously identify nonlinear dynamics and extract noise probability distributions from data. *Machine Learning: Science and Technology*, 3(1):015031, 2022.

[8] Fernando Lejarza and Michael Baldea. Data-driven discovery of the governing equations of dynamical systems via moving horizon optimization. *Scientific reports*, 12(1):11836, 2022.

[9] H Ribera, S Shirman, AV Nguyen, and NM Mangan. Model selection of chaotic systems from data with hidden variables using sparse data assimilation. *Chaos: An Interdisciplinary Journal of Nonlinear Science*, 32(6), 2022.

[10] Jeffrey M Hokanson, Gianluca Iaccarino, and Alireza Doostan. Simultaneous identification and denoising of dynamical systems. *SIAM Journal on Scientific Computing*, 45(4):A1413–A1437, 2023.

[11] National Aeronautics and Space Administration. Iss trajectory data, 2022. URL https://spotthestation.nasa.gov/trajectory_data.cfm.

[12] China Manned Space Agency. Orbital parameters of china's space station, 2024. URL https://www.cmse.gov.cn/gfgg/zgkjzgdcs/.

[13] Emil Simiu and Robert H Scanlan. *Wind effects on structures: fundamentals and applications to design*, volume 688. John Wiley New York, 1996.
